# Physiological relevance, localization and substrate specificity of the alternative (type II) mitochondrial NADH dehydrogenases of *Ogataea parapolymorpha*

**DOI:** 10.3389/fmicb.2024.1473869

**Published:** 2024-12-12

**Authors:** Hannes Juergens, Álvaro Mielgo-Gómez, Albert Godoy-Hernández, Jolanda ter Horst, Janine M. Nijenhuis, Duncan G. G. McMillan, Robert Mans

**Affiliations:** ^1^Department of Biotechnology, Delft University of Technology, Delft, Netherlands; ^2^School of Biological Sciences, University of Reading, Reading, United Kingdom

**Keywords:** mitochondria, NADH dehydrogensase, *Ogataea parapolymorpha*, bioenergetics, yeast engineering

## Abstract

Mitochondria from *Ogataea parapolymorpha* harbor a branched electron-transport chain containing a proton-pumping Complex I NADH dehydrogenase and three Type II NADH dehydrogenases (NDH-2). To investigate the physiological role, localization and substrate specificity of these enzymes, the growth of various NADH dehydrogenase knockout mutants was quantitatively characterized in shake-flask and chemostat cultures, followed by oxygen-uptake experiments with isolated mitochondria. NAD(P)H:quinone oxidoreduction of the three NDH-2 were individually assessed. Our findings reveal that the *O. parapolymorpha* respiratory chain contains an internal NADH-accepting NDH-2 (Ndh2-1/OpNdi1), at least one external NAD(P)H-accepting enzyme, and likely additional mechanisms for respiration-linked oxidation of cytosolic NADH. Metabolic regulation appears to prevent competition between OpNdi1 and Complex I for mitochondrial NADH. With the exception of OpNdi1, the respiratory chain of *O. parapolymorpha* exhibits metabolic redundancy and tolerates deletion of multiple NADH-dehydrogenase genes without compromising fully respiratory metabolism.

## Introduction

Dissimilation of glucose to carbon dioxide results in the generation of reducing equivalents, the primary type being in the form of NADH. For ongoing cell function, it is essential these are continuously (re)oxidized, a requirement intrinsically linked to the formation of ATP by the respiratory chain. In eukaryotes, glucose dissimilation and most of the NADH generation occurs in the cytosol during glycolysis and in the mitochondrial matrix by the combined action of the pyruvate-dehydrogenase complex and the tricarboxylic acid cycle. NADH cannot cross the inner mitochondrial membrane (von Jagow and Klingenberg, 1970), and as such needs to be (re)oxidized in the cellular compartment in which it was generated.

Fungal respiratory chains are typically branched and contain multiple entry points for electrons from NADH. While the large multi-subunit trans-membrane spanning type I NADH:quinone oxidoreductase (NDH-1 or Complex I) couples oxidation of mitochondrial NADH to the translocation of protons over the inner mitochondrial membrane, most fungi also possess monotopic alternative, type-II NADH dehydrogenases (NDH-2) that catalyze NADH:quinone oxidoreduction without proton translocation (Joseph-Horne et al., 2001). These ‘alternative NADH dehydrogenases’ are either peripheral membrane proteins, or monotopic membrane proteins (Boes et al., 2021) that are either associated or have a membrane anchoring helix attaching them to the inner mitochondrial membrane. They may differ in which side of the membrane they are located depending on cellular function. Their catalytic sites either face the mitochondrial matrix (‘internal’) where their catalytic activity overlaps with Complex I, or the intermembrane space (‘external’), allowing direct oxidation of cytosolic NADH (Antos-Krzeminska and Jarmuszkiewicz, 2019; Feng et al., 2012; Melo et al., 2004). *Saccharomyces cerevisiae* is an excellent example of an organism reliant on NDH-2. *S. cerevisiae* does not harbor a Complex I and relies solely on one internal and two external alternative NADH dehydrogenases as entry points for NADH-derived electrons into the respiratory chain (de Vries and Marres, 1987; Luttik et al., 1998). Besides NADH, some external fungal NDH-2, such as those from *Kluyveromyces lactis* (Tarrio et al., 2005; Tarrio et al., 2006) and *Neurospora crassa* (Carneiro et al., 2004; Carneiro et al., 2007; Melo et al., 2001), have also been reported to accept NADPH as substrate, either exclusively, or in addition to NADH. Based on the distribution of a key acidic residue (E272 in Ndi1 from *S. cerevisiae*), which has been proposed to prevent interaction with the phosphate group of NADPH (Iwata et al., 2012; Michalecka et al., 2004), it has been suggested that the majority of NDH-2 oxidize NADH rather than NADPH (Marreiros et al., 2016).

*Ogataea parapolymorpha* (formerly *Hansenula polymorpha*) is a methylotrophic, thermotolerant, Crabtree-negative yeast that is characterized by its rapid aerobic growth (Levine and Cooney, 1973; Juergens et al., 2018). It has a branched respiratory chain that contains one type I NADH dehydrogenase (Ndh1) and three putative type-II NAD(P)H dehydrogenases (referred to as Ndh2-1, Ndh2-2 and Ndh2-3 in this study) with unknown substrate specificity and unknown orientation on the inner mitochondrial membrane (Juergens et al., 2020). Interestingly, in the presence of excess glucose and oxygen, removal of Ndh1 from *O. parapolymorpha* does not result in a reduction of the maximum specific growth rate or biomass yield in batch cultures (Juergens et al., 2020). This implies that Ndh1 is disposable under these conditions. In aerobic glucose-limited cultures, elimination of Ndh1 resulted in mutant strain which exhibited a 16% lower biomass yield while it maintained a fully respiratory metabolism (Juergens et al., 2020). These phenotypes suggested that *O. parapolymorpha* harbors at least one internal alternative NADH dehydrogenase capable of compensating for loss of Ndh1, albeit at a lower efficiency of respiration-coupled ATP production due to a lack of protons pumped. Such an internal NADH dehydrogenase also would likely be responsible for (re)oxidation of mitochondrial NADH under fast-growing glucose-excess conditions, especially since NDH-2 appear to be suited to catalyze the high rates of NAD^+^ regeneration required to sustain a high glycolytic flux in the absence of fermentation (Godoy-Hernandez et al., 2019).

The aim of this study was to investigate the physiological roles of the three putative alternative NAD(P)H dehydrogenases of *O. parapolymorpha* with a special focus on determining whether this yeast indeed possesses an internal alternative NADH dehydrogenase capable of functionally substituting Ndh1. To this end, the aerobic growth characteristics of various *O. parapolymorpha* NAD(P)H-dehydrogenase mutant strains (*Δndh2-1*, *Δndh2-2* and *Δndh2-3*) were investigated in glucose grown batch and chemostat cultures. To determine localization and substrate specificity of the alternative dehydrogenases, substrate-dependent oxygen-uptake rates of isolated mitochondria from wild type and mutant strains were measured, and activity of the individual membrane-bound dehydrogenases with NADH and NADPH was assessed.

## Results

### Disruption of Ndh2-1 leads to a reduction of specific growth rate and a Crabtree-positive phenotype in *Ogataea parapolymorpha*

To investigate the contribution to respiration of the three putative alternative NAD(P)H dehydrogenases, strains IMD003, IMD004 and IMD005 were constructed, which harbored a disrupted version of the structural gene for Ndh2-1, Ndh2-2 or Ndh2-3, respectively (*Δndh2-1*, *Δndh2-2* and *Δndh2-3*). Ndh2-1 is a peripheral membrane protein, while Ndh2-2 or Ndh2-3 are monotopic membrane proteins containing a single predicted transmembrane helix (see Supplementary Figure S1). The physiology of these mutant strains was then assessed in aerobic shake-flask batch cultures in the presence of excess glucose (2 g L^−1^). The high specific rate of oxygen uptake by Crabtree-negative yeasts can lead to oxygen limitation in shake-flask cultures resulting in respiro-fermentative metabolism (Kiers et al., 1998). However, under the cultivation conditions in this study, the wild-type *O. parapolymorpha* strain CBS11895 grows with a full respiratory phenotype. It has a comparable specific growth rate as previously described in fully aerobic bioreactors, indicating that oxygen limitation did not occur (Table 1; Juergens et al., 2018).

**Table 1:** Physiology of wild-type *O. parapolymorpha* CBS11895 and congenic mutant strains in aerobic shake-flask cultures grown at 30°C in synthetic medium with urea as nitrogen- and glucose (2 g L^−1^) as carbon source.

Strain (genotype)	Specific growth rate (h^−1^)	Physiology
CBS11895 (*wild type*)	0.36 ± 0.01	Respiratory
IMD003 (*Δndh2-1*)	0.30 ± 0.01	0.31 ± 0.02 mol_EtOH_ mol_Glucose_^−1^
IMD004 (*Δndh2-2*)	0.34 ± 0.01	Respiratory
IMD005 (*Δndh2-3*)	0.35 ± 0.01	Respiratory
IMX1978 (*Δndh2-2 Δndh2-3*)	0.32 ± 0.00	Respiratory
IMX2017 (*Δndh2-1 Δndh2-2 Δndh2-3*)	0.30 ± 0.00	0.29 ± 0.02 mol_EtOH_ mol_Glucose_^−1^
IMX2197 (*Δnubm Δndh2-2 Δndh2-3*)	0.31 ± 0.00	Respiratory

Data are presented as mean ± mean absolute deviation from at least two independent shake-flask cultures. Specific growth rates and ethanol yield were calculated from the exponential growth phase on glucose.

In shake-flask cultures, the specific growth rate of mutant strain IMD005 (*Δndh2-3*) did not differ significantly from that of the wild-type strain CBS11895, whereas strains IMD003 (*Δndh2-1*) and IMD004 (*Δndh2-2*) exhibited 17 and 6% lower specific growth rates, respectively (Table 1). In cultures of strains CBS11895, IMD004 and IMD005 no fermentation products were detected, indicating that a disruption of either *NDH2-2* or *NDH2-3* did not impede aerobic respiratory metabolism. In contrast, strain IMD003 exhibited a Crabtree-positive phenotype, producing 0.31 ± 0.02 mol of ethanol for each mol of glucose consumed.

We then combined disruptions in *NDH2-2* and *NDH2-3*, resulting in strain IMX1978, which exhibited a slightly lower specific growth rate than the strains with individually disrupted *NDH* genes, but still maintained respiratory metabolism (Table 1). An additional disruption in strain IMX1978 of *NUBM*, which encodes an essential 51 kDA subunit of Ndh1 (Fecke et al., 1994), also did not result in detectable ethanol production in the resulting strain IMX2197 (*Δnubm(ndh1) Δndh2-2 Δndh2-3*). Collectively, these results demonstrate that Ndh2-1 alone is capable of supporting NAD(P)H turnover requirements in *O. parapolymorpha* for fully respiratory growth in a glucose grown batch culture.

### Oxygen consumption studies with mitochondria from wild-type *Ogataea parapolymorpha* and *NDH-2* deletion mutants confirm internal orientation of Ndh2-1

Strain IMX2017, in which all three *NDH2* genes were disrupted, exhibited a phenotype similar to that of strain IMD003 (*Δndh2-1*), displaying a 17% lower specific growth rate than observed for the wild-type strain CBS11895, and an ethanol yield similar to IMD003 (Table 1). Since yeast respiratory chains typically possess either none, or a single internal alternative NAD(P)H dehydrogenase (Antos-Krzeminska and Jarmuszkiewicz, 2019), and respiratory Complex I is not physiologically relevant under these conditions in *O. parapolymorpha* (Juergens et al., 2020), these data support the hypothesis that Ndh2-1 has an internal orientation (i.e., facing the mitochondrial matrix).

Oxygen-uptake rates of isolated mitochondria using a compartmentalized substrate approach has been previously used to determine the orientation of yeast mitochondrial NAD(P)H dehydrogenases (Luttik et al., 1998; Tarrio et al., 2005; Gonzalez-Barroso et al., 2006). To obtain *O. parapolymorpha* mitochondria, we adapted a protocol for isolation of mitochondria from glucose-limited *S. cerevisiae* cultures (Luttik et al., 1998). Initial isolations from wild-type cells grown in glucose-limited chemostat cultures resulted in well-coupled *O. parapolymorpha* mitochondria with a ‘respiratory control ratio’ (RCR) of 3.6 when assayed with endogenous NADH (generated in the mitochondrial matrix by addition of pyruvate and malate). However, the same preparations exhibited rapid, uncoupled oxygen uptake when exposed to methanol or ethanol. Methanol and ethanol dependent oxygen uptake rates were approximately 4-fold and 2.5-fold faster, respectively, than ADP-stimulated respiration of endogenous NADH. (M)ethanol-dependent oxygen uptake indicated a minor contamination of the mitochondrial preparations of these glucose-de-repressed cultures with peroxisomes containing methanol oxidase (MOX) (Egli et al., 1980; Yurimoto et al., 2011). A similar contamination was previously observed in mitochondrial preparations from the methylotrophic yeast *Pichia pastoris* (Gonzalez-Barroso et al., 2006). In an attempt to obtain mitochondrial preparations devoid of MOX activity, mitochondria were also isolated from cells grown under MOX-repressing conditions in glucose-grown batch- or nitrogen-limited chemostat cultures (Egli and Quayle, 1986; Ravin et al., 2013). Mitochondrial isolations from cells grown under these conditions did not consume oxygen in the presence of methanol, but they exhibited RCRs close to 1 when assayed with endogenous NADH, indicating uncoupled preparations (data not shown). In light of these findings, mitochondria isolated from glucose-limited chemostat cultures were used for respiration studies, and interference of the oxygen-uptake measurements by MOX activity was minimized by using reaction mixtures and substrates devoid of alcoholic solvents (see Methods section).

To test the hypothesis that the NADH binding site of Ndh2-1 is oriented toward the mitochondrial matrix, oxygen uptake was measured in the presence of endogenous and exogenous NADH using mitochondria isolated from wild type *O. parapolymorpha* (CBS11895), from strains possessing only Ndh1 (IMX2017; *Δndh2-1 Δndh2-2 Δndh2-3*) or only Ndh2-1 (IMX2197; *Δnubm; Δndh2-2; Δndh2-3*), henceforth known as ‘respiration-linked NAD(P)H dehydrogenases’ (Table 2). These strains were of interest because in aerobic glucose-limited chemostat cultures at a dilution rate of 0.1 h^−1^, all three strains exhibited a fully respiratory metabolism (see below, Table 3), indicating a functional respiratory chain capable of (re)oxidation of both cytosolic and mitochondrial NADH pools.

**Table 2:** Substrate-dependent rates of oxygen uptake by mitochondria from wild-type *O. parapolymorpha* (CBS11895) and mutants possessing only Ndh1 (IMX2017) or Ndh2-1 (IMX2197) as known respiration-linked NAD(P)H dehydrogenases.

Substrate	All Ndh enzymes (CBS11895)			Ndh1 only (IMX2017: *Δndh2-1; Δndh2-2; Δndh2-3*)		Ndh2-1 only (IMX2197: Δ*nubm; Δndh2-2; Δndh2-3*)	
	O_2_ uptake rate	RCR	KCN inhibition	O_2_ uptake rate	RCR	O_2_ uptake rate	RCR
Pyruvate + malate	0.15 ± 0.02	3.6 ± 0.4	92%	0.14 ± 0.00	3.2 ± 0.4	0.18 ± 0.00	2.7 ± 0.6
Pyruvate + malate (rotenone)	0.03 ± 0.00	1.6 ± 0.0	N.D.	0.04 ± 0.00	1.9 ± 0.1	0.16 ± 0.00	3.1 ± 0.5
NADH	0.33 ± 0.05	2.5 ± 0.1	94%	0.04 ± 0.00	1.4 ± 0.2	0.04 ± 0.01	1.1 ± 0.0
NADPH	0.09 ± 0.01	1.1 ± 0.0	85%	0.03 ± 0.00	2.9 ± 0.3	0.01 ± 0.00	1.4 ± 0.2

Mitochondria were isolated from cells grown in aerobic, glucose-limited chemostat cultures at a dilution rate (D) of 0.1 h^−1^ and assayed at 30°C, pH 7.0. Oxygen uptake in μmol O_2_ (mg protein)^−1^ min^−1^ was determined in the presence of 0.25 mM ADP. Respiratory control ratio (RCR) values represent the ratio of oxygen uptake rates in the presence and absence (prior to addition) of ADP. For tests with rotenone (50 μM), mitochondria were pre-incubated with this inhibitor at assay conditions prior to substrate addition. Oxygen-uptake rates and RCRs are presented as mean ± standard deviation of measurements with mitochondria from at least two independent chemostat cultures for each strain. Tests with KCN (1 mM) were performed with mitochondria from a single, representative isolation of strain CBS11895. N.D, Not determined.

**Table 3:** Physiology of *Ogataea parapolymorpha* strains CBS11895 (wild type), IMX2017 (Ndh1 only) and IMX2197 (Ndh2-1 only) in aerobic, glucose-limited chemostat cultures grown at a dilution of 0.1 h^−1^, 30°C and pH 5.

Strain	CBS11895* wild type	IMX2017 only Ndh1	IMX2197 only Ndh2-1
Actual dilution rate (h^−1^)	0.10 ± 0.00	0.10 ± 0.00	0.10 ± 0.00
Reservoir glucose (g L^−1^)	7.49 ± 0.00	7.36 ± 0.01	9.04 ± 0.01
Y_X/S_ (g biomass [g glucose]^−1^)	0.51 ± 0.00	0.52 ± 0.00	0.44 ± 0.01
Y_X/O2_ (g biomass [g O_2_]^−1^)	1.35 ± 0.05	1.36 ± 0.00	0.94 ± 0.01
RQ (−)	1.05 ± 0.01	1.05 ± 0.00	1.04 ± 0.00
q_Glucose_ (mmol [g biomass]^−1^ h^−1^)	−1.08 ± 0.03	−1.07 ± 0.01	−1.28 ± 0.00
q_CO2_ (mmol [g biomass]^−1^ h^−1^)	2.44 ± 0.07	2.40 ± 0.01	3.50 ± 0.00
q_O2_ (mmol [g biomass]^−1^ h^−1^)	−2.32 ± 0.08	−2.30 ± 0.01	−3.38 ± 0.00
C_X_ (g biomass L^−1^)	3.84 ± 0.08	3.83 ± 0.04	4.01 ± 0.05
Carbon recovery (%)	99.3 ± 1.2	100.1 ± 0.8	98.8 ± 0.8

Data are presented as mean ± mean absolute deviation from two independent replicates. Carbon recoveries were calculated based on a biomass carbon content of 48% (w/w). Symbols: Y_X/S_ and Y_X/O2_ = yield of biomass dry weight on glucose and oxygen, respectively; RQ = respiratory quotient; q_Glucose_, q_CO2_ and q_O2_ represent biomass-specific uptake/production rates of glucose, CO_2_ and O_2_, respectively; C_X_ represents biomass dry weight concentration. *Data for CBS11895 were reproduced from Juergens, Niemeijer (Juergens et al., 2018).

Mitochondria isolated from wild type *O. parapolymorpha* (CBS11895) readily consumed oxygen in the presence of endogenous and exogenous NADH (Table 2). RCRs for endogenous NADH (3.6 ± 0.4) and exogenous NADH (2.5 ± 0.1) indicated that the observed respiration linked NADH oxidation is due to the activity of separate internal and external NADH dehydrogenases, and not the result of physically compromised mitochondria. Furthermore, the near-complete inhibition of oxygen utilization by the cytochrome c oxidase inhibitor cyanide (~92 and ~94% effective in the presence of endogenous and exogenous NADH, respectively) strongly suggests that oxygen consumption was dependent on an intact oxidative membrane-bound electron transport chain capable of maintaining a proton motive force sufficient for ATP synthesis. In contrast, mitochondria from strains IMX2017 (Ndh1 only) and IMX2197 (Ndh2-1 only) both exhibited 88% lower oxygen consumption rates in the presence of exogenous NADH, suggesting that mitochondria from these strains do not possess any notable external NADH dehydrogenase activity characteristic of a type-II NADH dehydrogenase.

Mitochondria from strains IMX2017 (Ndh1 only) and IMX2197 (Ndh2-1 only) when assayed with endogenous NADH exhibited similar coupled oxygen-uptake rates to mitochondria isolated from the wild-type strain CBS11895 (Table 2). Presence of rotenone, the Ndh1-specific inhibitor, strongly decreased oxygen-uptake rates with endogenous NADH of mitochondria from the strains CBS11895 and IMX2017 (by 80 and 71%, respectively), while for strain IMX2197 no significant rotenone inhibition was observed. These observations demonstrate that Ndh2-1 is indeed internally oriented and able to completely take over the role of Ndh1 in NADH oxidation in *Δnubm* mutants under aerobic, glucose-limited conditions.

When mitochondria isolated from the wild-type strain *O. parapolymorpha* CBS11895 were assayed with exogenous NADPH, oxygen consumption was detected at a rate of 0.09 ± 0.01 μmol O_2_ (mg protein)^−1^ min^−1^, substantially lower than the rate obtained with exogenous NADH (0.33 ± 0.05 μmol O_2_ (mg protein)^−1^ min^−1^, Table 2). NADPH-dependent oxygen consumption was not significantly coupled (RCR of 1.1). However, it was largely inhibited by cyanide (~85%), indicating that NADPH oxidation did not occur via a soluble enzyme but by an external NADPH-accepting dehydrogenase that transferred electrons from NADPH into the mitochondrial respiratory chain. In mitochondria isolated from strains IMX2017 and IMX2197 and challenged with cyanide, exogenous NADPH oxidation activity was reduced by 3- and 9-fold, respectively, strongly suggesting that Ndh2-2 and/or Ndh2-3 is responsible for the NADPH oxidation observed in CBS11895 mitochondria.

### *Ogataea parapolymorpha* mutants with linearized respiratory chains exhibit respiratory physiology in glucose-limited chemostat cultures

In aerobic glucose-limited chemostat cultures grown at a dilution rate of 0.1 h^−1^, both strain IMX2017 (Ndh1 only*; Δndh2-1 Δndh2-2 Δndh2-3*) and strain IMX2197 (Ndh2-1 only; *Δnubm Δndh2-2 Δndh2-3*) exhibited essentially the same respiratory phenotype as the wild-type strain CBS11895. Despite the deletion of one respiration-linked NAD(P)H dehydrogenase, both mutant strains grew without detectable formation of fermentation products. Moreover, an oxidative respiratory quotient close to 1 was observed and the carbon contained in the glucose feed could be completely recovered as biomass and carbon dioxide (Table 3). IMX2017 exhibited a biomass yield of 0.52 g biomass (g glucose)^−1^, which is not significantly different from that found with wild type CBS11895 (0.51 g biomass (g glucose)^−1^). In contrast, the biomass yield of strain IMX2197 was reduced by ~15% compared to that of the two other strains. This reduced biomass yield is consistent with a less efficient oxidative respiratory chain, using internal, non-proton pumping Ndh2-1 instead of the proton-pumping Ndh1. Accordingly, IMX2197 exhibited an approximately ~30% lower biomass yield on oxygen, with correspondingly higher biomass-specific rates of oxygen consumption and carbon-dioxide production.

### The *Ogataea parapolymorpha* NDH2s oxidize NADH but not NADPH when expressed in *Escherichia coli* membranes

The oxygen-consumption experiments indicated that mitochondria from *O. parapolymorpha* poorly couple oxidation of exogenous NADPH to oxygen consumption via the aerobic respiratory chain. In principle, any external Ndh-2 could be responsible for this activity. Upon closer examination of the putative amino acid sequence of *O. parapolymorpha* Ndh2-3, an uncharged residue (Q365) instead of a negatively charged (E272 in *S. cerevisiae* Ndi1) is present within the substrate-binding domain (Supplementary Figure S2). This residue has been suggested to be involved in determining NADH/NADPH specificity due to interaction with the phosphate group of NADPH (Iwata et al., 2012; Michalecka et al., 2004).

To assess the ability of the *O. parapolymorpha* Ndh-2 to catalyze the oxidation of NADH and/or NADPH, *NDH2-1*, *NDH2-2* and *NDH2-3* were individually overexpressed in *Escherichia coli*, following a strategy previously applied to an *NDH-2* (*NDI1*) from *S. cerevisiae* (Kitajima-Ihara and Yagi, 1998) and *Caldalkalibacillus thermarum* (Godoy-Hernandez et al., 2019; Godoy-Hernandez and McMillan, 2021). Since respiration-linked NADPH-dehydrogenase activity has not been reported for *E. coli*, this host was regarded as especially suitable to assess NADPH-dehydrogenase activity of heterologously expressed genes.

**Figure 1:**
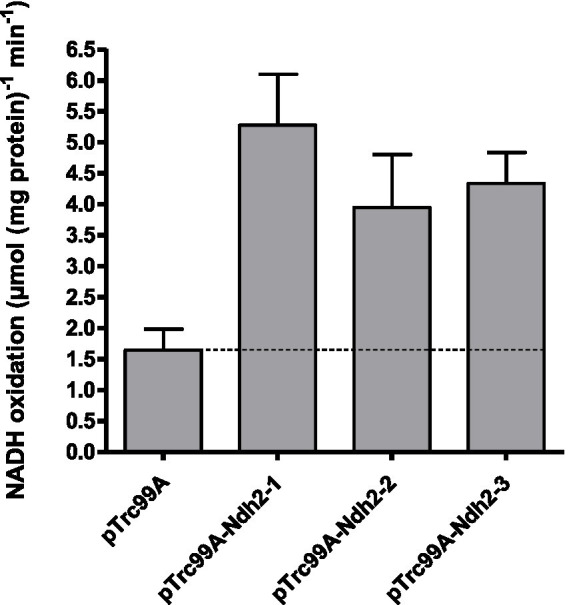
NADH oxidation by *E. coli* membranes isolated from strains overexpressing individual *O. parapolymorpha NDH-2*. Control measurements were done with membranes isolated from a strain carrying an empty overexpression plasmid (pTrc99A). Assays were performed with a membrane protein concentration of 10 μg mL^−1^ at 37°C, with 200 μM NADH and 100 μM ubiquinone-1. Data is presented as mean ± standard deviation of triplicate measurements.

In spectrophotometric assays at pH 7.4, expression of each of the three *O. parapolymorpha NDH-2* led to 2.4–3.2-fold higher NADH-oxidation rates than observed with membranes isolated from the parental *E. coli* strain (Figure 1). This activity indicated successful overexpression and localization to the *E. coli* membrane, and confirmed that the three *O. parapolymorpha* Ndh-2 are indeed NADH:quinone oxidoreductases. When NADPH was added as substrate to the same membrane preparations, no detectable NADPH-oxidation was measurable, indicating that none of the three *O. parapolymorpha* Ndh-2 can effectively utilize NADPH under the conditions tested (Supplementary Table S1). Since NAD(P)H oxidation by Ndh-2 can depend on pH (Melo et al., 2001; Nantapong et al., 2005), NADH and NADPH oxidation measurements were repeated at pH 5.5 and 8.0. These different pH values did not influence NADH oxidation rates relative to rates measured at pH 7.4 (Student’s t test, *p* > 0.05), and did not stimulate NADPH utilization by either endogenous *E. coli* respiratory enzymes or *O. parapolymorpha* Ndh-2 (Supplementary Table S1). Finally, oxidative NAD(P)H catalysis by Ndh2-3 overexpressed in *E. coli* was also tested in the presence of calcium, as Ndh2-3 contains a putative EF-hand suggesting a calcium binding domain which could potentially regulate catalytic behavior (Supplementary Figure S3; Melo et al., 2001). However, the presence of 5 mM Ca^2+^ did not significantly affect rates of NADH oxidation by Ndh2-3 overexpression membranes at pH 5.5 and 7.4 (24% reduction at pH 8.0) and did not enable NADPH utilization at pH 5.5, 7.4 or 8.0 (Supplementary Table S1).

Taken together, these data conclusively show that when assessed in *E. coli* membranes, the three NDH2s from *O. parapolymorpha* can efficiently oxidize NADH at a range of pH levels (pH 5.5–8.0), but do not catalyze the oxidation of NADPH.

## Discussion

### NAD(P)H dehydrogenases in the respiratory chain of *Ogataea parapolymorpha*

In this study, the *O. parapolymorpha* gene HPODL_02792 (*NDH2-1*) was demonstrated to encode a mitochondrial ‘internal’ alternative NADH dehydrogenase. To achieve consistent nomenclature with other fungal species, we suggest naming this gene *OpNDI1* (NADH dehydrogenase, internal). The closely related yeast *O. polymorpha* likely also possesses an internal alternative NADH dehydrogenase, as its genome (Riley et al., 2016) encodes a protein (OGAPODRAFT_15258) that exhibits 98% sequence identity with OpNdi1.

Mitochondria isolated from *O. parapolymorpha* also oxidized externally supplied NADH, indicating that either one, or both of the remaining alternative dehydrogenases, Ndh2-2 and Ndh2-3, are facing the intermembrane space, rather than the mitochondrial matrix. Coexistence of more than one internal alternative dehydrogenase has been described (Rasmusson et al., 2004), but since the fungi and yeasts so far characterized either possess one or no such enzymes, both Ndh2-2 and Ndh2-3 appear to be externally oriented. The internal orientation of Ndh2-1/OpNdi1 has now been established in this study.

Wild-type *O. parapolymorpha* mitochondria also oxidized NADPH via the respiratory chain as demonstrated by near-complete inhibition of this activity by cyanide. While growing on glucose the production of NADPH can be balanced with biosynthetic requirements, assimilation of other carbon sources such as gluconate result in a surplus of NADPH in yeast (Bruinenberg et al., 1983a), making the ability to respire NADPH a beneficial trait. The oxygen-uptake activity with NADPH was essentially uncoupled (RCR of 1.1), which was unexpected, as addition of ADP should relieve the backpressure of the proton gradient, especially since the same mitochondrial preparations exhibited well-coupled oxygen uptake in the presence of NADH. Mitochondria isolated from glucose-limited chemostat cultures of *K. lactis* (D = 0.1 h^−1^) similarly exhibited uncoupled oxygen uptake in the presence of NADPH (Overkamp et al., 2002), while mitochondria from lactate- and glucose-grown batch cultures of the same yeast exhibited RCRs of 2.3 and 1.3 for NADPH, respectively (Tarrio et al., 2006). With mitochondria isolated from glucose-limited chemostats of *Candida utilis*, the RCRs of NADPH oxidation were found to vary as a function of the dilution rate, with cultures grown at *D* = 0.05–0.1 h^−1^ exhibiting an RCR of ~1.9 while cultures grown at *D* = 0.2–0.4 h^−1^ exhibited a lower RCR of ~1.2 (Bruinenberg et al., 1985a). In agreement with our study, these studies reported inhibition of NADPH oxidation by cyanide and/or antimycin A, indicating that the degree of oxidative coupling of NADPH respiration by yeast mitochondria is dependent on the utilized substrate and/or the growth condition employed, which might also be the case in *O. parapolymorpha*.

The heterologous expression of *O. parapolymorpha NDH-2* in *E. coli* showed that NADPH oxidation of the *O. parapolymorpha* mitochondria is not likely to have occurred via these Ndh-2, since all three *O. parapolymorpha* Ndh-2 exclusively utilized NADH when assayed within *E. coli* membranes. Based on their protein sequences, we originally speculated Ndh2-3 to be the most likely candidate to accept NADPH, as it contains the uncharged residue (Q365) proposed to permit NADPH utilization (Michalecka et al., 2004). There are indeed NADPH-utilizing NDH2s with this uncharged residue such as Nde1 from *N. crassa* (Supplementary Figure S1) or plant enzyme St-NBD1 (Michalecka et al., 2004), and mutation of this exact residue has been exploited to alter substrate specificity from NADH to co-utilization of NADH and NADPH in a bacterial NDH2 (Desplats et al., 2007). However, Nde2 from *N. crassa* and Nde1 from *K. lactis* do not contain the uncharged residue but still accept both substrates (Supplementary Figure S2), indicating that a charged amino acid in this position does not strictly prevent NADPH utilization and that Ndh-2 substrate specificity cannot be accurately predicted by a single residue. Another possibility is that Ndh2-3 could be post-translationally modified in *O. parapolymorpha,* in this instance, a post-translational modification (PTM) could theoretically tune the activity of Ndh2-3 toward NADPH. However, this possibility is unlikely and no enzyme has been reported to date to have a PTM to modify active site substrate specificity, moreover, we could also not detect any yeast PTM sites near the nucleotide binding sites using the YAAM database (Ledesma et al., 2018). In contrast to the NADPH utilizing Nde1 from *N. crassa*, where NADPH oxidation is highly affected by calcium (Melo et al., 2001), the putative calcium binding domains of Ndh2-3 from *O. parapolymorpha* and Nde2 from *K. lactis* are poorly conserved, and apparently lost their regulatory function, as presence of calcium did not appear to have an effect on activity of either enzyme (Tarrio et al., 2006). Collectively, this supports the notion that the three type-II Ndh from *O. parapolymorpha* are unlikely to be responsible for the observed NADPH oxidation activity, indicating the presence of another unidentified enzyme.

### Mechanisms independent of Ndh-2 for turnover of cytosolic NADH

Mitochondria from strains IMX2017 (Ndh1 only; *Δndh2-1 Δndh2-2 Δndh2-3*) and IMX2197 (Ndh2-1 only; *Δnubm Δndh2-2 Δndh2-3*) did not oxidize external NADH, but both strains exhibited a fully respiratory phenotype in glucose-limited chemostat cultures at *D* = 0.1 h^−1^. Since both strains only contain a NADH dehydrogenase that can accept electrons from NADH in the mitochondrial matrix (Ndh1 and Ndh2-1/OpNdi1, respectively), mechanisms other than the external alternative NADH dehydrogenases are capable of respiring cytosolic NADH in these strains must be present. One candidate would be the ‘Gut2/Gpd shuttle’, consisting of mitochondrial glycerol-3-phosphate dehydrogenase (Gut2), which indirectly respires cytosolic NADH in combination with cytosolic NAD-dependent glycerol 3-phosphate dehydrogenase (Gpd) (Bakker et al., 2001). When grown on glycerol, *O. parapolymorpha* exhibits highly increased glycerol-kinase activity, indicating that glycerol assimilation occurs via the phosphorylative pathway. This suggests that Gut2 is functional in this yeast (Tani and Yamada, 1987). Furthermore, Gut2 activity was demonstrated in the closely related yeast *O. polymorpha* (de Koning et al., 1987). In *S. cerevisiae*, the metabolic function of Gut2 overlaps with the external Ndh-2 (Larsson et al., 1998; Overkamp et al., 2000), and yeast mitochondria isolated from glucose-grown cultures of various yeast species have been demonstrated to respire glycerol-3-phosphate with similar specific oxygen uptake rates as NADH (Luttik et al., 1998; Gonzalez-Barroso et al., 2006; Overkamp et al., 2002).

While *S. cerevisiae* lacking Gut2 in addition to its two external NADH dehydrogenases produces large amounts of glycerol under aerobic, glucose-limited conditions (Palmieri et al., 2006), an *O. parapolymorpha* mutant lacking Gut2 in addition to the three Ndh-2 (*Δndh2-1 Δndh2-2 Δndh2-3 Δgut2*) exhibited fully respiratory physiology under identical conditions (Supplementary Table S2). The absence of byproduct formation indicates that at least one additional, unknown mechanism for coupling the oxidation of cytosolic NADH to mitochondrial respiration is present in *O. parapolymorpha*. Such a mechanism could, for example, involve a shuttle for import of NADH equivalents into the mitochondrial matrix, where oxidation by Complex I and/or OpNdi1 can occur. For *S. cerevisiae*, several suitable mechanisms have been demonstrated or proposed, such as the malate-oxaloacetate and malate–aspartate shuttles, mitochondrial oxidation of ethanol produced in the cytosol, or, assuming a high cytosolic NADH/NAD^+^ ratio, an inward-directed ethanol-acetaldehyde shuttle (Bakker et al., 2001; Palmieri et al., 2006). Based on sequence homology with *S. cerevisiae*, key proteins for these mechanisms are present in *O. parapolymorpha* (Supplementary Table S3), however their operation and physiological relevance has not been confirmed by functional analysis studies.

### Physiological relevance of branched respiratory chains

Respiratory chains of plants, fungi and some protists are branched (Antos-Krzeminska and Jarmuszkiewicz, 2019; Rasmusson et al., 2008), and, as also demonstrated in this study for *O. parapolymorpha*, can exhibit a metabolic redundancy for respiration-linked NADH oxidation, minimizing the physiological effect of loss of individual or multiple NADH dehydrogenases (Carneiro et al., 2004; Overkamp et al., 2000; Fromm et al., 2016). In general, this redundancy and the exact physiological role of the alternative NADH dehydrogenases are still poorly understood. In *O. parapolymorpha*, OpNdi1 appears to have a unique metabolic function as it is the only NADH dehydrogenase strictly required to sustain fast respiratory growth under glucose excess conditions, demonstrating that limiting respiratory capacity by disruption of a single NADH dehydrogenase can elicit the Crabtree effect in a Crabtree-negative yeast. Similarly, the overexpression of a single ‘Gal4-like’ transcription factor has been reported to convert Crabtree-negative *P. pastoris* into a Crabtree-positive yeast (Ata et al., 2018). However, since formation of ethanol and CO_2_ from glucose by alcoholic fermentation is redox-neutral and reduced by-products such as glycerol were not detected in fermenting cultures of IMD003 (*Δndh2-1*) and IMX2017 (*Δndh2-1 Δndh2-2 Δndh2-3*), (re)oxidation of mitochondrial NADH from oxidative sugar metabolism must still occur via respiration. While Ndh1 has been demonstrated to be physiologically irrelevant under these glucose excess conditions in wild-type *O. parapolymorpha* CBS11895 (Juergens et al., 2020), based on the available data its contribution to oxidation of mitochondrial NADH cannot be excluded in these strains. Alternatively, if export of NADH equivalents from the mitochondrial matrix is possible in this yeast, NADH could also be oxidized in the cytosol, for example by external NADH dehydrogenase(s) in IMD003 (*Δndh2-1*) or the Gut2/Gpd shuttle in IMX2017 (Ndh1 only).

A previous study with sub-mitochondrial particles harvested from stationary-phase cultures of *O. polymorpha* found that most NADH oxidation occurred via Ndh-2 and only ~10% via Ndh1 (Bridges et al., 2009). Similarly, in our experiments with mitochondria isolated from wild-type *O. parapolymorpha* grown in glucose-limited chemostat cultures, only ~30% of the total specific NADH oxidation activity could be attributed to Ndh1. However, due to the saturating substrate concentrations used in these types of experiments, they allow only for a limited interpretation of the actual physiological relevance of the respective enzymes. *In vivo*, competition of Complex I and OpNdi1 for NADH likely occurs if both systems are expressed at the same time, and indeed some fungal species appear to co-utilize Complex I and internal Ndh-2 (Fecke et al., 1994; Prömper et al., 1993; Voulgaris et al., 2012). With mitochondria isolated from wild-type *O. parapolymorpha* strain CBS11895, the presence of 50 μM rotenone could not fully abolish internal NADH oxidation. However, as strain IMX2017 (Ndh1 only), exhibited a similar partial inhibition of oxygen uptake by rotenone, the uninhibited activity in CBS11895 was likely not caused by OpNdi1 but instead by incomplete inhibition of *O. parapolymorpha* Ndh1. Comparative studies with sub-mitochondrial particles have demonstrated that rotenone inhibits Ndh1 from yeasts less strongly than the *Bos taurus* enzyme, requiring 50 μM rotenone to achieve 96% inhibition of NADH oxidation activity by the *P. pastoris* Ndh1 (Bridges et al., 2009). It is conceivable that Ndh1 from O*. parapolymorpha* is even more resistant to rotenone, explaining the observed partial inhibition in strains CBS11895 (wild type) and IMX2017 (Ndh1 only). In addition, OpNdi1 was not detected in the proteome of aerobic, glucose-limited cultures of CBS11895 (Juergens et al., 2020), and the observed identical biomass yields of strains CBS11895 and IMX2017 are consistent with a situation in which essentially all mitochondrial NADH is (re)oxidized by Ndh1 in wild-type *O. parapolymorpha* under these conditions. These observations indicate that oxidation of mitochondrial NADH is strictly separated between Ndh1 and OpNdi1 in *O. parapolymorpha* under conditions of glucose limitation and glucose excess, respectively. Nevertheless, OpNdi1 is able to fully support respiratory growth in the absence of a functional Complex I, as previously hypothesized (Juergens et al., 2020).

### Concluding remarks

In this study we show that the Crabtree-negative yeast *O. parapolymorpha* contains both NDH1- and NDH2-type NADH dehydrogenases for respiration of NADH in the mitochondrial matrix but limits their utilization to conditions of carbon limitation and carbon excess, respectively. Furthermore, we find that the respiratory chain of *O. parapolymorpha* can tolerate multiple deletions without compromising respiratory metabolism, offering insight into its flexible nature and opportunities for metabolic (redox) engineering, for example to increase cytosolic NADH availability, in this industrially relevant yeast. Finally, the phenotype elicited by disruption of OpNdi1 demonstrates that limiting respiratory capacity by a single mutation in an NADH dehydrogenase can result in overflow metabolism and convert *O. parapolymorpha* into a yeast with a Crabtree-positive phenotype.

## Materials and methods

### Yeast strains and maintenance

The *O. parapolymorpha* strains used in this study are derived from the wild-type strain CBS11895 (DL-1; ATCC26012) and are described in Table 4. For construction and maintenance, yeast strains were grown in an Innova shaker incubator (New Brunswick Scientific, Edison, NJ, USA) set to 30°C and 200 rpm, in 500 mL shake flasks containing 100 mL heat-sterilized (120°C for 20 min) YPD medium (10 g L^−1^ Bacto yeast extract, 20 g L^−1^ Bacto peptone, 20 g L^−1^ glucose, demineralized water). Solid medium was prepared by addition of 2% (w/v) agar. Frozen stock cultures were prepared from exponentially growing shake-flask cultures by addition of glycerol to a final concentration of 30% (v/v), and aseptically stored in 1 mL aliquots at −80°C.

**Table 4:** *O. parapolymorpha* (*Hansenula polymorpha*) strains used in this study.

Strain	Genotype	Origin
CBS11895	Wild type *O. parapolymorpha* (DL-1)	CBS-KNAW^a^
IMD003	*ndh2-1^∆266–269^*	This study
IMD004	*ndh2-2^A289AT^*	This study
IMD005	*ndh2-3^∆505–515^*	This study
IMX1945	*Δndh2-2::AgTEF1p-hph-AgTEF1t*	This study
IMX1978	*Δndh2-2::AgTEF1p-hph-AgTEF1t Δndh2-3::AaTEF1p-NatR-ScPHO5t*	This study
IMX2197	*Δndh2-2::AgTEF1p-hph-AgTEF1t Δndh2-3::AaTEF1p-NatR-ScPHO5t Δnubm::AgTEF1p-pat-AgTEF1t*	This study
IMX2017	*Δndh2-2::AgTEF1p-hph-AgTEF1t Δndh2-3::AaTEF1p-NatR-ScPHO5t Δndh2-1::ScTEF1p-kanR-ScTDH1t*	This study
IMX2167	*Δndh2-2::AgTEF1p-hph-AgTEF1t Δndh2-3::AaTEF1p-NatR-ScPHO5t Δndh2-1::ScTEF1p-kanR-ScTDH1t Δgut2::AgTEF1p-pat-AgTEF1t*	This study

a CBS11895 was obtained from the CBS-KNAW fungal collection (Westerdijk Fungal Biodiversity Institute, Utrecht, The Netherlands).

### Plasmid construction

All plasmids used in this study are described in Table 5. Plasmids pUD546, pUD547 and pUD548 were *de novo* synthesized by GeneArt (Thermo Fisher Scientific, Waltham, MA, USA) and contained synthetic guide RNA (gRNA) constructs with spacer sequences (5′-3′) ‘CCTGATGTAAATATACGCTG’, ‘AAGAAGAACATTGTTATTCT’, and ‘GTTATTCTGGGTTCCGGCTG’, respectively. Cas9/gRNA co-expression plasmids pUDP019, pUDP020 and pUDP021 were constructed using pUD546, pUD547 and pUD548, respectively, by integration into pUDP002 via BsaI-mediated ‘Golden Gate’ assembly (Engler et al., 2008) as described previously (Juergens et al., 2018), and verified by digestion with PdmI.

**Table 5:** Plasmids used in this study.

Name	Relevant characteristics	Origin
pUD546	^BsaI^HH-gRNA*_NDH2-1_*-HDV^BsaI^	GeneArt
pUD547	^BsaI^HH-gRNA*_NDH2-2_*-HDV^BsaI^	GeneArt
pUD548	^BsaI^HH-gRNA*_NDH2-3_*-HDV^BsaI^	GeneArt
pUDP002	*panARS (OPT) AgTEF1p-hph-AgTEF1t ScTDH3p* ^BsaI BsaI^ *ScCYC1t AaTEF1p-Spcas9-ScPHO5t*	Juergens et al. (2018)
pUDP019	*panARS (OPT) AgTEF1p-hph-AgTEF1t ScTDH3p*-HH-gRNA*_NDH2-1_*-HDV-*ScCYC1t AaTEF1p-Spcas9-ScPHO5t*	This study
pUDP020	*panARS (OPT) AgTEF1p-hph-AgTEF1t ScTDH3p*-HH-gRNA*_NDH2-2_*-HDV-*ScCYC1t AaTEF1p-Spcas9-ScPHO5t*	This study
pUDP021	*panARS (OPT) AgTEF1p-hph-AgTEF1t ScTDH3p*-HH-gRNA*_NDH2-3_*-HDV-*ScCYC1t AaTEF1p-Spcas9-ScPHO5t*	This study
pUD602	Template for plasmid backbone (*ori ampR*)	Juergens et al. (2018)
pYTK013	Template for *ScTEF1p* promoter	Lee et al. (2015)
pYTK056	Template for *ScTDH1t* terminator	Lee et al. (2015)
pYTK077	Template for *kanR* ORF	Lee et al. (2015)
pYTK078	Template for *NatR* ORF	Lee et al. (2015)
pAG31	Template for *AgTEF1p-pat-AgTEF1t* cassette	Goldstein and McCusker (1999)
pUD740	Template for *ScTEF1p-kanR-ScTDH1t* cassette	This study
pUD801	HRS*_NDH2-1_*-*ScTEF1p-kanR-ScTDH1t*-HRS*_NDH2-1_*	This study
pUD802	HRS*_NDH2-2_*-*AgTEF1p-hph-AgTEF1t-*HRS*_NDH2-2_*	This study
pUD803	HRS*_NDH2-3_*-*AaTEF1p-NatR-ScPHO5t*-HRS*_NDH2-3_*	This study
pUD1035	HRS*_GUT2_*-*AgTEF1p-pat-AgTEF1t*-HRS*_GUT2_*	This study
pUD1036	HRS*_NUBM_*-*AgTEF1p-pat-AgTEF1t*-HRS*_NUBM_*	This study
pTrc99A	*ori ampR trp/lac*-MCS-*rrnB*	Amann et al. (1988)
pTrc99A-NDH2-1	*ori ampR trp/lac*-*NDH2-1*-*rrnB*	This study
pTrc99A-NDH2-2	*ori ampR trp/lac*-*NDH2-2*-*rrnB*	This study
pTrc99A-NDH2-3	*ori ampR trp/lac*-*NDH2-3*-*rrnB*	This study

Restriction enzyme sites are indicated in superscript, and genes targeted by gRNAs or homologous recombination are indicated in subscript. Aa, *Arxula adeninivorans*; Sp, *Streptococcus pyogenes*; Ag, *Ashbya gossypii*; Sc, *Saccharomyces cerevisiae*; HH, hammerhead ribozyme; HDV, hepatitis delta virus ribozyme; MCS, multiple cloning site; HRS, homologous recombination sequence.

Construction of pUD740 was done via ‘Gibson assembly’ (Gibson et al., 2009) from the following PCR-amplified fragments: the *ScTEF1* promoter from pYTK013 (primers 12099 + 12100), the *kanR* G418 resistance marker ORF from pYTK077 (primers 12097 + 12098), the *ScTDH1* terminator from pYTK056 (primers 12095 + 12096), the *AaTEF1p-Spcas9-ScPHO5t* expression cassette from pUDP002 (primers 10426 + 10427), upstream (primers 12093 + 12094) and downstream (primers 12101 + 12102) homologous recombination sequences of the *OpKU70* locus from CBS11895 genomic DNA and the plasmid backbone from pUD602 (primers 12103 + 12104).

Plasmids pUD801, pUD802, pUD803, pUD1035 and pUD1036 carrying subcloned homology-flanked marker cassettes for split-marker deletion were constructed by Gibson assembly from various PCR-amplified fragments. For the construction of pUD801 these fragments were: the *ScTEF1p-kanR-ScTDH1t* G418 resistance marker cassette from pUD740 (primers 12100 + 4377), upstream (primers 14385 + 14386) and downstream (primers 14387 + 14388) homologous recombination sequences of the *NDH2-1* locus from CBS11895 genomic DNA and the plasmid backbone from pUD602 (primers 12103 + 12104). For pUD802 these fragments were: the *AgTEF1p-hph-AgTEF1t* hygromycin resistance marker cassette from pUDP002 (primers 11065 + 11133), upstream (primers 14389 + 14390) and downstream (primers 14391 + 14392) homologous recombination sequences of the *NDH2-2* locus from CBS11895 genomic DNA and the plasmid backbone from pUD602 (primers 12103 + 12104). For pUD803 these fragments were: the *AaTEF1* promoter from pUDP002 (primers 10426 + 14383), the *NatR* nourseothricin resistance marker ORF from pYTK078 (primers 14381 + 14382), the *ScPHO5* terminator from pUDP002 (primers 10427 + 14384), upstream (primers 14393 + 14394) and downstream (primers 14395 + 14396) homologous recombination sequences of the *NDH2-3* locus from CBS11895 genomic DNA and the plasmid backbone from pUD602 (primers 12103 + 12104). For pUD1035 these fragments were: the *AgTEF1p-pat-AgTEF1t* phosphinothricin resistance marker cassette from pAG31 (primers 3242 + 8439), upstream (primers 14803 + 14804) and downstream (primers 14805 + 14806) homologous recombination sequences for *GUT2* from CBS11895 genomic DNA and the plasmid backbone from pUD602 (primers 12103 + 12104). For pUD1036 these fragments were: the *AgTEF1p-pat-AgTEF1t* phosphinothricin resistance marker cassette from pAG31 (primers 3242 + 8439), upstream (primers 14807 + 14808) and downstream (primers 14809 + 14810) homologous recombination sequences for *NUBM* from CBS11895 genomic DNA and the plasmid backbone from pUD602 (primers 12103 + 12104). Correct insertion and presence of the homology-flanked marker cassettes in the constructed plasmids was verified by restriction digest and diagnostic PCR using PvuI + NdeI and primer sets 2908 + 12616 and 1642 + 3983 (pUD801), PvuI + PsiI and primer sets 2457 + 12616 and 1642 + 1781 (pUD802), PvuI + KpnI and primer sets 10459 + 12616 and 1642 + 10458 (pUD803) and PvuI + NcoI and primer sets 1409 + 12616 and 1642 + 4662 (pUD1035 & pUD1036).

Plasmids pTrc99A-NDH2-1, pTrc99A-NDH2-2 and pTrc99A-NDH2-3 were constructed by restriction/ligation cloning. The ORFs encoding *NDH2-1* (HPODL_02792), *NDH2-2* (HPODL_00256) and *NDH2-3* (HPODL_02018) were PCR-amplified from CBS11895 genomic DNA using primer sets 14929 + 14931, 16075 + 16076 and 16077 + 16078, respectively. Amplification using these primer sets added a 5′ NcoI site, a GS-flanked 6-HIS tag (‘GSHHHHHHGS’) directly after the start codon and a 3′ XmaI site to all three ORFs. Furthermore, amplification of *NDH2-1* omitted the first 24 amino acids after the start codon, as they could be unambiguously identified as mitochondrial targeting sequence by MitoFates (Fukasawa et al., 2015). PCR amplicons were then digested with NcoI and XmaI and cloned into NcoI/XmaI-digested pTrc99A using T4 DNA ligase (New England Biolabs, Ipswich, MA, USA).

### Yeast strain construction

*Ogataea parapolymorpha* strains were transformed via electroporation of freshly prepared electrocompetent cells as described previously (Juergens et al., 2018). Depending on the selection marker, mutants were selected on solid YPD medium supplemented with 200 μg mL^−1^ G418, 300 μg mL^−1^ hygromycin B or 100 μg mL^−1^ nourseothricin, or on solid synthetic medium (SM) supplemented with 20 g L^−1^ glucose and 200 μg mL^−1^ bialaphos (SanBio, Uden, The Netherlands). SM was prepared according to Verduyn, Postma (Verduyn et al., 1992) and autoclaved at 120°C for 20 min. Glucose and vitamins (Verduyn et al., 1992) were prepared separately and filter-sterilized (vitamins) or heat-sterilized at 110°C for 20 min (glucose).

Strains IMD003, IMD004 and IMD005 with disrupted versions of genes *NDH2-1* (HPODL_02792), *NDH2-2* (HPODL_00256) and *NDH2-3* (HPODL_02018), respectively, were constructed using the pUDP CRISPR/Cas9 system described previously (Juergens et al., 2018). Wild type strain CBS11895 was transformed with pUDP019, pUDP020 or pUDP021 targeting *NDH2-1* (after base part 269 out of 1,614), *NDH2-2* (after base pair 290 out of 1,671) and *NDH2-3* (after base pair 515 out of 2,097), respectively, and subjected to the prolonged liquid incubation protocol as described previously for deletion of *OpADE2* and *OpKU80* (Juergens et al., 2018). Randomly picked colonies were then subjected to PCR amplification of the *NDH2-1*, *NDH2-2* and *NDH2-3* locus using primer sets 10742 + 10743, 10744 + 10745 and 10746 + 10747, respectively, followed by Sanger sequencing (Baseclear, Leiden, The Netherlands) to identify mutant transformants harboring a frame-shifting indel at the respective gRNA target sites. Three mutants with either a deletion of base pairs 226–229 of *NDH2-1*, an additional thymine nucleotide between position 289 and 290 of *NDH2-2* or a deletion of base pairs 505–515 of *NDH2-3* were identified, restreaked three times subsequently on non-selective YPD medium to remove the pUDP plasmids, and renamed IMD003, IMD004 and IMD005, respectively.

Strains IMX1945, IMX1978, IMX2017, IMX2197 and IMX2167 were constructed using a split-marker deletion approach (Fairhead et al., 1996), with ~480 bp of internal (marker recombination) and ~480 bp of external (genome recombination) homology. To preserve the promoter and terminator sequences of neighboring genes and limit interference of their expression, a minimum of 800 bp preceding and 300 bp succeeding adjacent ORFs were kept unaffected by the deletions. IMX1945 was constructed from wild type *O. parapolymorpha* strain CBS11895 by deletion of *NDH2-2* with a *hyg* resistance cassette using pUD802, IMX1978 was constructed from IMX1945 by additional deletion of *NDH2-3* with a *NatR* resistance cassette using pUD803, IMX2017 was constructed from IMX1978 by additional deletion of *NDH2-1* with a *kanR* resistance cassette using pUD801, IMX2197 was constructed from IMX1978 by additional deletion of *NUBM* (HPODL_04625) (Juergens et al., 2020) with a *pat* resistance cassette using pUD1036, and IMX2167 was constructed from IMX2017 by additional deletion of *GUT2* (HPODL_00581) with a *pat* resistance cassette using pUD1035. For the split-marker deletion of *NDH2-1*, *NDH2-2*, *NDH2-3*, *NUBM* and *GUT2*, the two overlapping fragments for transformation were PCR-amplified from pUD801 using primer sets 6816 + 14397 and 12565 + 14398, from pUD802 using primer sets 14399 + 14400 and 14401 + 14402, from pUD803 using primer sets 14403 + 14404 and 14405 + 14406, from pUD1036 using primer sets 15884 + 15885 and 15886 + 15887, and from pUD1035 using primer sets 14811 + 15885 and 14812 + 15886, respectively. Prior to transformation, the amplified fragments were gel-purified and, in case DNA amounts were too low for transformation, used as template for another PCR amplification using the same primers followed by PCR purification. For each transformation, a total of ~1 μg purified DNA (both fragments equimolar) in a maximum volume of 4 μL was transformed to 40 μL of fresh electrocompetent cells as described above, with the exception that after electroporation the cell suspensions were recovered in 1 mL YPD for 3 h at 30°C before plating onto selective medium. Additionally, after YPD recovery, cells transformed with the *pat* resistance marker were washed once by centrifugation and resuspension in sterile demineralized water before selective plating. Selection plates were typically incubated for 3 days at 30°C before assessment of the correct replacement of the target genes with the resistance markers via diagnostic PCR using primer sets 14465 + 14466, 14465 + 4047 and 2653 + 14466 for *Δndh2-1::kanR*, primer sets 14467 + 14468, 14467 + 7864 and 8411 + 14468 for *Δndh2-2::hph*, primer sets 14469 + 14470, 14469 + 11197 and 11202 + 14470 for *Δndh2-3::NatR*, primer sets 15630 + 15631, 15630 + 15885 and 15886 + 15631 for *Δnubm::pat* and primer sets 15628 + 15629, 15628 + 15885 and 15886 + 15629 for *ΔGUT2::pat*. Single colonies that contained the desired genotype(s) were re-streaked once on selective medium, followed by two re-streaks on non-selective YPD medium before stocking.

### Molecular biology

PCR amplification for cloning and construction was performed with Phusion High Fidelity Polymerase (Thermo Fisher Scientific) using PAGE-purified oligonucleotide primers (Sigma-Aldrich, St. Louis, MO, USA) according to manufacturer’s recommendations, with the exception that a final primer concentration of 0.2 μM was used. Diagnostic PCR was done using DreamTaq polymerase (Thermo Fisher Scientific) and desalted primers (Sigma-Aldrich). The primers used in this study are shown in Supplementary Table S4. Genomic DNA of yeast colonies was isolated using the LiAc-sodium dodecyl sulfate method (Lõoke et al., 2011) or the YeaStar Genomic DNA kit (Zymo Research, Irvine, CA, USA). DNA fragments obtained by PCR were separated by gel electrophoresis. Gel-purification was carried out using the Zymoclean Gel DNA Recovery Kit (Zymo Research). PCR purification was performed using the GenElute PCR Clean-Up Kit (Sigma-Aldrich). Gibson assembly was done using the NEBuilder HiFi DNA Assembly Master Mix (New England Biolabs) with purified DNA fragments according to manufacturer’s recommendations, with the exception that reaction volume was down-scaled to 5–10 μL. DNA fragments that were PCR-amplified from a template harboring the same bacterial resistance marker as the construct to be Gibson-assembled were subjected to DpnI treatment prior to PCR cleanup. Restriction digest was performed using FastDigest enzymes (Thermo Fisher Scientific) or High Fidelity (HF) restriction endonucleases (New England Biolabs, Ipswich, MA, USA) according to the manufacturer’s instructions. *Escherichia coli* strains XL1-blue and DH5α were used for plasmid transformation, amplification and storage. Plasmid isolation from *E. coli* was done using the GenElute Plasmid Miniprep Kit (Sigma-Aldrich) or the Monarch Plasmid Miniprep Kit (New England Biolabs).

### Multiple sequence alignment and domain prediction

Alignment of Ndh-2 protein sequences was done using MUSCLE^1^ (Edgar, 2004) and visualized using Jalview (Waterhouse et al., 2009). Orientation and substrate specificity of other fungal Ndh-2 were taken from: *K. lactis* (Tarrio et al., 2005; Tarrio et al., 2006), *N. crassa* (Carneiro et al., 2004; Carneiro et al., 2007; Melo et al., 2001; Duarte et al., 2003), *S. cerevisiae* (de Vries and Marres, 1987; Luttik et al., 1998; Small and McAlister-Henn, 1998; Van Urk et al., 1989), and *Y. lipolytica* (Kerscher et al., 1999). Prediction of the putative EF-hand calcium binding domain in Ndh-2 sequences was done by Motif Scan^2^ using PROSITE profiles (Sigrist et al., 2010). Prediction of transmembrane helicies was performed using TMHMM—2.0 (Krogh et al., 2001).

### Shake-flask cultivation

Shake-flask growth experiments were performed with synthetic medium with urea as nitrogen source to avoid fast acidification (Luttik et al., 2000), set to an initial pH of 5.0 with KOH. Cultures were grown in 500 mL round-bottom shake flasks filled with 50 mL medium. Cultures were grown with 2 g L^−1^ glucose as sole carbon source and were inoculated with mid-exponential precultures (washed once with sterile demineralized water) to an initial OD_660_ of 0.3. Precultures were grown under the same conditions and in the same medium, but with an initial glucose concentration of 5 g L^−1^. Shake flasks were continuously shaken during sampling to prevent oxygen limitation. Physiological parameters were calculated from at least five samples taken during the exponential growth phase. Calculated ethanol yields were not corrected for evaporation.

### Chemostat cultivation

Chemostat cultivation was performed as described previously (Juergens et al., 2020) using SM with the addition of 0.15 g L^−1^ Pluronic 6100 PE antifoaming agent (BASF, Ludwigshafen, Germany) and glucose (7.5 or 9 g L^−1^) as sole carbon source. SM was prepared according to Verduyn, Postma (Lee et al., 2015) as described above. Bioreactors were inoculated with exponentially growing shake flask cultures (SM with 20 g L^−1^ glucose). Chemostat cultivation was performed in 2-L benchtop bioreactors (Applikon, Delft, The Netherlands) with a working volume of 1.0 L which was maintained by an electrical level sensor that controlled the effluent pump. The dilution rate was set by maintaining a constant medium inflow rate. Cultures were sparged with dried, compressed air (0.5 vvm) and stirred at 800 rpm. Temperature was maintained at 30°C and pH was controlled at 5.0 by automatic addition of a 2 M KOH by an EZcontroller (Applikon). The exhaust gas was cooled with a condenser (2°C) and dried with a Perma Pure Dryer (Inacom Instruments, Veenendaal, the Netherlands) prior to online analysis of carbon dioxide and oxygen with a Rosemount NGA 2000 Analyzer (Emerson, St. Louis, MO, USA). Cultures were assumed to have reached steady state when, after a minimum of 5 volume changes, the oxygen-consumption rate, carbon-dioxide production rate and biomass concentration changed by less than 3% over two consecutive volume changes.

### Analytical methods

Optical density (OD) of yeast cultures was measured at 660 nm on a Jenway 7200 spectrophotometer (Jenway, Staffordshire, UK). OD of bacterial cultures was measured at 600 nm on Ultrospec 2100 pro (Amersham, Little Chalfont, UK). For biomass dry weight determination of yeast cultures, exactly 10 mL of culture broth was filtered over pre-dried and pre-weighed membrane filters (0.45 μm, Pall corporation, Ann Arbor, MI, USA), which were washed with demineralized water, dried in a microwave oven at 350 W for 20 min and weighed immediately (Postma et al., 1989). Samples were diluted with demineralized water prior to filtration to obtain a biomass dry weight concentration of approximately 2 g L^−1^. The exact dilution was calculated by weighing the amount of sample and diluent and assuming a density of 1 g mL^−1^ for both fractions. Concentrations of extracellular metabolites and putative alcoholic contaminants of NAD(P)H substrates were analyzed by high-performance liquid chromatography (HPLC) on an Agilent 1100 HPLC (Agilent Technologies, Santa Clara, CA, USA) with an Aminex HPX-87H ion-exchange column (BioRad, Veenendaal, The Netherlands) operated at 60°C with 5 mM H_2_SO_4_ as mobile phase at a flow rate of 0.6 mL min^−1^. For the determination of extracellular metabolites, 1 mL aliquots of culture broth were centrifuged for 3 min at 20,000 *g* and the supernatant was used for analysis. Protein concentrations of mitochondrial preparations were estimated by the Lowry method (Lowry et al., 1951), using dried bovine serum albumin (BSA, fatty acid-free, Sigma-Aldrich) as standard. Where necessary, protein determinations were corrected for BSA present in the mitochondrial preparations. Protein concentrations of *E. coli* membrane fractions were determined using a bicinchoninic acid (Smith et al., 1985) protein assay kit (Sigma-Aldrich) with BSA (Interchim, Montlucon, France) as standard.

### Isolation of mitochondrial fractions

Mitochondria were isolated from glucose-limited, aerobic chemostat cultures (*D* = 0.1 h^−1^) according to a procedure similar as described for *S. cerevisiae* (Luttik et al., 1998), based on the mild osmotic lysis method developed for *Candida utilis* (Bruinenberg et al., 1985b). Biomass (1.5 g dry weight) was harvested by centrifugation at 3,000 *g* for 4 min. The pellet was then resuspended by vortexing in 30 mL of Tris buffer (100 mM) containing 10 mM dithiothreitol (final buffer pH of 9.3) and incubated at 30°C for 10 min. Afterwards, the cells were washed twice with 30 mL buffer A (25 mM potassium phosphate, 2 M sorbitol, 1 mM MgCl_2_, 1 mM EDTA, pH 7.5) by centrifugation (4,000 *g*, 4 min) and resuspension (gentle vortexing). Then, cells were pelleted again by centrifugation (5,000 *g*, 8 min) and resuspended (gentle vortexing) in a total volume of 40 mL buffer A. 3.06 mg of zymolyase (from *Arthrobacter luteus*, 20,000 U g^−1^, AMS Biotechnology, Abingdon, UK) dissolved in 200 μL buffer A was added to the cell suspension, which was subsequently incubated at 30°C under gentle shaking for 60–90 min. Incubation time depended on the rate of spheroplast formation which was estimated based on the sensitivity to osmotic shock by 200-fold dilution in demineralized water as described previously (Bruinenberg et al., 1985b). Incubation was continued until osmotic resistance decreased to approx. 25% (see Supplementary Figure S4). During the zymolyase treatment, release of glucose-6-phosphate dehydrogenase activity from compromised cells was measured as described previously (Bruinenberg et al., 1985b; Bruinenberg et al., 1983b) and typically did not exceed 5% compared to a sonicated sample. After zymolyase treatment, all subsequent steps were carried out on ice or in a cooled (4°C) centrifuge. Spheroplasts were washed twice with 35 mL buffer A by centrifugation (4,400 *g*, 7 min) and resuspension (gentle shaking), followed by centrifugation (5,000 *g*, 6 min) and resuspension (gentle shaking) in a total volume of 10 mL buffer A. Subsequently, 30 mL of buffer B (25 mM potassium phosphate, 0.2 M sorbitol, 1 mM MgCl_2_, 1 mM EDTA, pH 7.5) was added dropwise to the spheroplast suspension over a timeframe of ~2–3 h, while it was slowly stirred with a magnetic stirrer bar. The spheroplast suspension was then subjected to two strokes in a cooled Potter-Elvehjem homogenizer (150 rpm, clearance 28 μm). After centrifugation (3,000 *g*, 10 min), the supernatant was separated from intact cells and debris and spun again (12,000 *g*, 10 min). The resulting pellet, containing the mitochondria, was resuspended in 2.5 mL of buffer C (25 mM potassium phosphate, 0.65 M sorbitol, 1 mM MgCl_2_, 1 mM EDTA, 1 mg mL^−1^ BSA (fatty acid-free, Sigma-Aldrich), pH 7.5) and kept on ice.

### Oxygen-uptake studies with mitochondrial preparations

Substrate-dependent oxygen consumption rates of mitochondria were determined polarographically at 30°C with a Clark-type oxygen electrode (McMillan et al., 2009). The assay mixture (4 mL) contained 25 mM potassium phosphate buffer (pH 7.0), 5 mM MgCl_2_, and 0.65 M sorbitol. Reactions were started with ethanol (5 mM), methanol (5 mM), L-malate + pyruvate (both 5 mM, adjusted to pH 7.0 with KOH), 0.25 mM NADH (Prozomix, Haltwhistle, UK) or 0.75 mM NADPH (Oriental Yeast Co., Tokyo, Japan). While some commercial preparations of NADH and NADPH are contaminated with ethanol (Luttik et al., 1998; Overkamp et al., 2002; Patchett and Jones, 1986), no ethanol (or methanol) was detected via HPLC analysis in freshly prepared, concentrated (100 mM) solutions of the NADH and NADPH used in this study (detection limit: ethanol 1 mM; methanol 5 mM). Oxygen uptake rates were calculated based on a dissolved oxygen concentration of 236 μM in air-saturated water at 30°C. Respiratory control values were determined by adding 0.25 mM ADP (Chance and Williams, 1956). For tests with rotenone (50 μM), a concentrated stock solution (20 mM in DMSO) was freshly prepared directly before the respective assays and kept at room temperature. Mitochondria were pre-incubated in the presence of rotenone for 5 min at assay conditions prior to substrate addition. Preincubation with equivalent amounts of DMSO without rotenone did not measurably affect oxygen uptake rates. Tests with KCN (1 mM) were conducted with a concentrated stock solution (200 mM, in 100 mM NaOH), which was added to mitochondria during ADP-stimulated respiration (state III). Addition of equivalent amounts of NaOH without KCN affected oxygen uptake rates by less than 15%.

### Overexpression of *Ogataea parapolymorpha NDH-2* in *Escherichia coli*

Overexpression and purification strategy were based on previous literature (Godoy-Hernandez et al., 2019). *Escherichia coli* BL21(DE3) cells were transformed with plasmids pTrc99A, pTrc99A-Ndh2-1, pTrc99A-Ndh2-2, and pTrc99A-Ndh2-3. Correct transformants were pre-cultured (37°C, 180 rpm) for 16 h in lysogeny broth with 100 μg mL^−1^ ampicillin. These precultures were used to inoculate 500 mL apple flasks with 100 mL 2xYT medium (16 g L^−1^ tryptone, 10 g L^−1^ yeast extract, 5 g L^−1^ NaCl) supplemented with 20 g L^−1^ glucose and 100 μg mL^−1^ ampicillin and grown under the same conditions. Once cultures had reached an OD_600_ of 0.5, NDH2 overexpression was induced using 1 mM isopropyl β-D-1-thiogalactopyranoside (IPTG, Sigma Aldrich), followed by growth for an additional 5 h at 30°C and 180 rpm. Afterwards, cells were harvested by centrifugation (7,000 *g*, 10 min) and washed with 25 mL buffer W1 (50 mM Tris–HCl, pH 8.0, 2 mM MgCl_2_). Cell pellets were then resuspended in buffer W1 (at 4 mL per g wet weight), containing 0.1 mM PMSF (Sigma-Aldrich) and 0.1 mg mL^−1^ bovine pancreatic DNase (New England Biolabs), and incubated 10 min at room temperature. Cells were then disrupted using a cell disruptor (Constant Systems Ltd., Daventry, UK) at 1.38 kbar. Unbroken cells and cell debris were removed by centrifugation (10,000 *g*, 10 min). The membrane fraction was isolated from the cell lysate by ultracentrifugation (180,000 *g*, 45 min, 4°C), and the resulting membrane pellet was resuspended in buffer W1 and stored at −80°C.

### *In vitro* NAD(P)H dehydrogenase activity tests with Ndh-2

NAD(P)H:quinone oxidoreductase activity was measured using a spectrophotometric assay as previously described (Godoy-Hernandez et al., 2019; Godoy-Hernandez and McMillan, 2021). Activity was monitored spectrophotometrically using a modified Cary 60 UV/Vis Spectrophotometer (Agilent Technologies), following the oxidation of NADH or NADPH at 340 nm in the presence of ubiquinone-1 (UQ-1, Sigma-Aldrich) at 37°C. Membrane preparations (10 μg protein mL^−1^) and UQ-1 (100 mM) were added to pre-warmed reaction buffer (final volume 2 mL) in a 1 cm path length cuvette and incubated for 30 s. Depending on the pH, the reaction buffer consisted of (i) 50 mM Tris–HCl (pH 8.0), 150 mM NaCl, (ii) 25 mM MES + 25 mM MOPS (pH 7.4), 150 mM NaCl or (iii) 25 mM MES + 25 mM MOPS (pH 5.5), 150 mM NaCl. NADH or NADPH (200 μM at pH 8.0 and 7.4, 100 μM at pH 5.5) were added to the mixture to initiate the reaction. An extinction coefficient of 6.3 mM^−1^ cm^−1^ was used to calculate NAD(P)H concentration. For tests with calcium, 5 mM CaCl_2_ was added to the assay after 1 min of reaction time had elapsed.

## Data Availability

The raw data supporting the conclusions of this article will be made available by the authors without undue reservation.

## Footnotes

^1^ https://www.ebi.ac.uk/Tools/msa/muscle/

^2^ https://myhits.isb-sib.ch/cgi-bin/motif_scan
