# Supplementary material for: Physiological relevance, localization and substrate specificity of the alternative (type II) mitochondrial NADH dehydrogenases of Ogataea parapolymorpha
Source: Front Microbiol. 2024 Dec 12;15:1473869. doi: 10.3389/fmicb.2024.1473869 (PMC11670749; doi:10.3389/fmicb.2024.1473869)
Supplement: Supplementary file 1 [file Data_Sheet_1.docx]

**Physiological relevance, localization and substrate specificity of the alternative (type II) mitochondrial NADH dehydrogenases of *Ogataea parapolymorpha***

Hannes Juergens^1^, Álvaro Mielgo-Gómez^1^, Albert Godoy-Hernández^1^, Jolanda ter Horst^1^, Janine M. Nijenhuis^1^, Duncan G. G. McMillan^1,2*^ and Robert Mans^1*^

^1^Department of Biotechnology, Delft University of Technology, Delft, The Netherlands

^2^School of Biological Sciences, University of Reading, Reading, United Kingdom

*Corresponding authors: Robert Mans, [r.mans@tudelft.nl](mailto:r.mans@tudelft.nl) and Duncan McMillan, [d.g.g.mcmillan@tudelft.nl](mailto:d.g.g.mcmillan@tudelft.nl) OR d.g.g.mcmillan@reading.ac.uk

Running title: Function of *O. parapolymorpha* type II NADH dehydrogenases.

Keywords: *Mitochondria; NADH Dehydrogensase; Ogataea parapolymorpha; Bioenergetics; Yeast engineering.*

**Supplementary material**

**
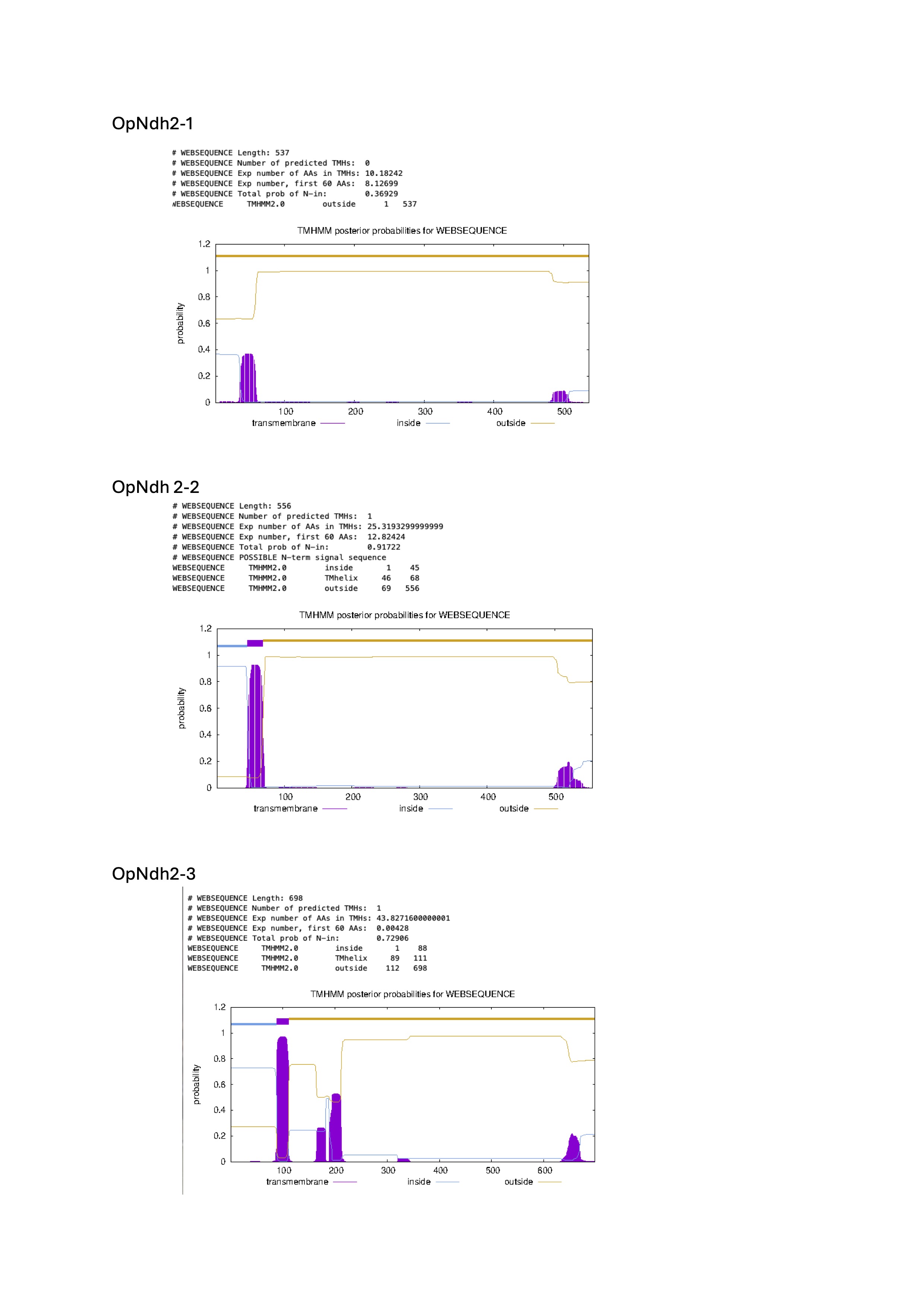
Figure S1:** Prediction of transmembrane helicies using TMHMM (67) in *Ogataea parapolymorpha* Ndh2.1, Ndh2.2 and Ndh2.3. Both OpNdh2.2 and OpNdh2.3 contain 1 predicted transmembrane helix, where OpNdh2.1 lacks one.

Figure S2: Section of MUSCLE sequence alignment (60) of fungal/yeast NDH2 sequences, visualized by Jalview using Clustalx residue colors. Black boxes denote (left) conserved GxGxxGxE motif of second dinucleotide (substrate) binding domain and (right) residue affecting NADH/NADPH substrate specificity. Orientation, either I (internal) or E (external) and substrate utilization, NADH, NADPH or NAD(P)H (= both) are depicted for characterized fungal NDH2s. *Kl*, *Kluyveromyces lactis*, *Nc*, *Neurospora crassa*, *Op*, *Ogataea parapolymorpha*, *Sc*, *Saccharomyces cerevisiae*, *Yl*, *Yarrowia lipolytica*. E272


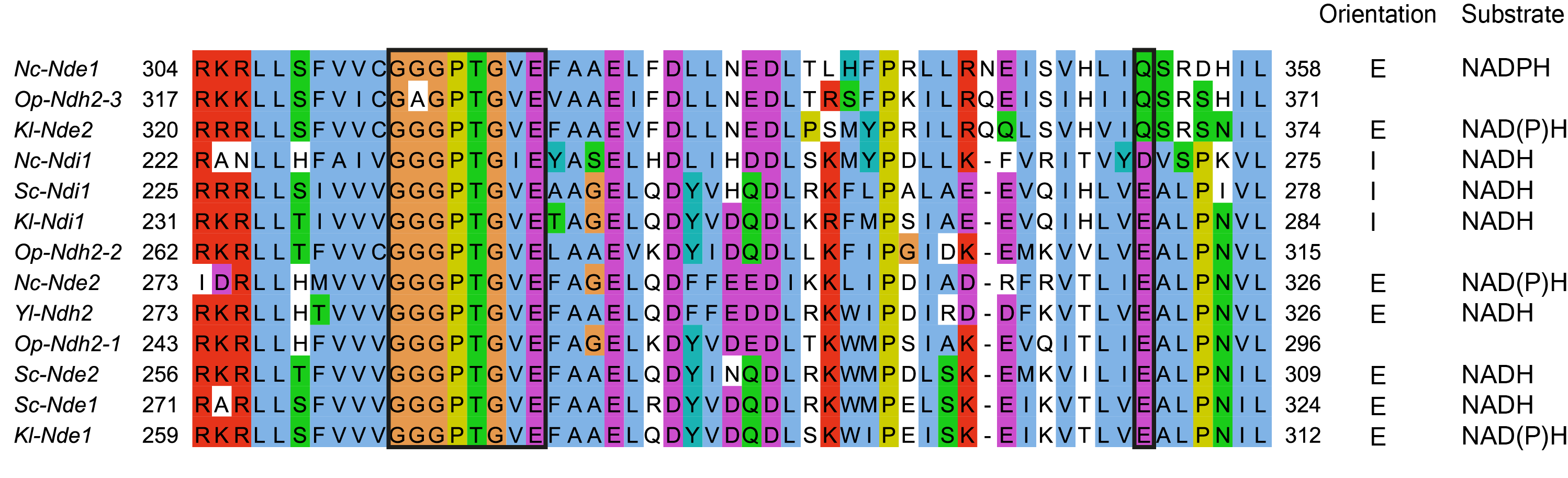


Figure S3: Section of MUSCLE multiple sequence alignment (60) of fungal/yeast *NDH2* sequences, visualized by Jalview using Clustalx residue colors. The black box denotes a (putative) EF-hand calcium binding domain as predicted by Motif Scan. *Kl*, *Kluyveromyces lactis*, *Nc*, *Neurospora crassa*, *Op*, *Ogataea parapolymorpha*, *Sc*, *Saccharomyces cerevisiae*, Yl, *Yarrowia lipolytica*.


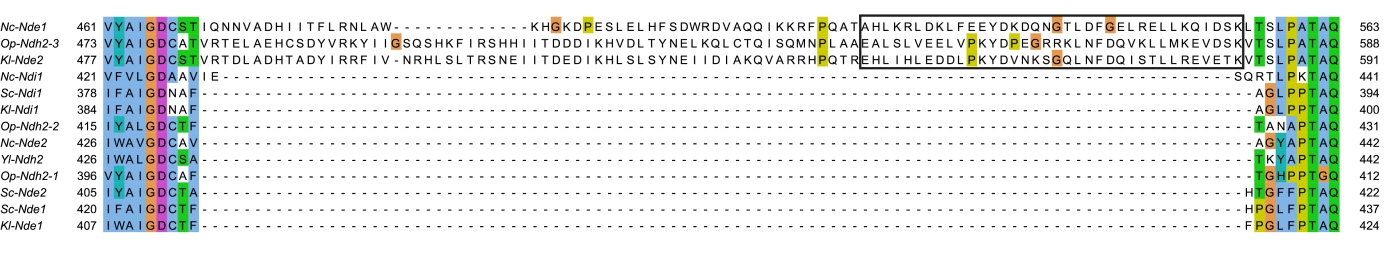


Figure S4: Proportion of osmotically insensitive cells of *O. parapolymorpha* strains CBS11895 (triangles), IMX2017 (diamonds) and IMX2197 (squares) during zymolyase treatment as determined by dilution in demineralized water. Data is presented as mean ± standard deviation from at least 2 independent replicates and was normalized to initial OD for each strain individually.

Table S1: NAD(P)H oxidation (µmol (mg protein)^-1^ min^-1^) by *E. coli* membranes isolated from strains overexpressing individual *O. parapolymorpha* NDH2s. Control measurements were done with membranes isolated from a strain carrying an empty overexpression plasmid (pTrc99A). Assays were performed with a membrane protein concentration of 10 µg mL^-1^ at 37°C, with 200 µM (pH 7.4 and 8) or 100 µM (pH 5.5) NAD(P)H and 100 µM ubiquinone-1. For tests with calcium, 5 mM CaCl was used. Data is presented as mean ± standard deviation of at least duplicate measurements. ^a^Single measurement.

|  | NADH | | | NADPH | | |
| --- | --- | --- | --- | --- | --- | --- |
|  | pH 5.5 | pH 7.4 | pH 8.0 | pH 5.5 | pH 7.4 | pH 8.0 |
| Control  pTrc99A | 1.64 ± 0.34 | 1.64 ± 0.34 | 1.49 ± 0.11 | 0.03 ± 0.04 | 0.02 ± 0.03 | 0.18 ± 0.01 |
| Ndh2-1  pTrc99A-NDH2-1 | 4.47 ± 0.05 | 5.27 ± 0.82 | 4.58 ± 0.25 | < 0.01 | 0.03 ± 0.05 | 0.05 ± 0.05 |
| Ndh2-2  pTrc99A-NDH2-2 | 3.45 ± 0.13 | 3.95 ± 0.85 | 3.24 ± 0.94 | 0.06 ± 0.08 | 0.02 ± 0.04 | 0.09 ± 0.09 |
| Ndh2-3  pTrc99A-NDH2-3 | 3.77 ± 0.05 | 4.34 ± 0.50 | 4.70 ± 0.02 | 0.21 ± 0.16 | 0.01 ± 0.02 | 0.03 ± 0.04 |
| Control + Ca^2+^  pTrc99A | 1.43^a^ | 1.64 ± 0.12 | 1.64 ± 0.09 | 0.07 ± 0.05 | < 0.01 | 0.09 ± 0.02 |
| Ndh2-3 + Ca^2+^  pTrc99A-NDH2-3 | 3.08 ± 0.25 | 4.15 ± 0.69 | 3.57 ± 0.09 | 0.17 ± 0.16 | < 0.01 | < 0.01 |

Table S2: Physiology of *Ogataea parapolymorpha* strain IMX2167 in aerobic, glucose-limited chemostat cultures grown at a dilution rate of 0.1 h^-1^ at 30°C and pH 5. Data are presented as mean ± mean absolute deviation from two independent replicates. Carbon recoveries were calculated based on a biomass carbon content of 48% (w/w). Symbols: Y_X/S_ and Y_X/O2_ = yield of biomass dry weight on glucose and oxygen, respectively; RQ = respiratory quotient; q_Glucose_, q_CO2_ and q_O2_ represent biomass-specific uptake/production rates of glucose, CO_2_ and O_2_, respectively; C_X_ represents biomass dry weight concentration.

| **Strain** | **IMX2167**  *Δndh2-1 Δndh2-2 Δndh2-3 Δgut2* |
| --- | --- |
| Actual dilution rate (h^-1^) | 0.10 ± 0.00 |
| Reservoir glucose (g L^-1^) | 7.61 ± 0.01 |
| Y_X/S_ (g biomass [g glucose]^-1^) | 0.52 ± 0.00 |
| Y_X/O2_ (g biomass [g O_2_]^-1^) | 1.44 ± 0.00 |
| RQ (-) | 1.05 ± 0.00 |
| q_Glucose_ (mmol [g biomass]^-1^ h^-1^) | -1.04 ± 0.00 |
| q_CO2_ (mmol [g biomass]^-1^ h^-1^) | 2.25 ± 0.01 |
| q_O2_ (mmol [g biomass]^-1^ h^-1^) | -2.14 ± 0.00 |
| C_X_ (g biomass L^-1^) | 3.97 ± 0.00 |
| Carbon recovery (%) | 98.7 ± 0.3 |

Table S3: *S. cerevisiae* proteins (proposed to be) involved in ethanol-acetaldehyde, malate-oxaloacetate and malate-aspartate shuttle NADH shuttles, and corresponding ortholog proteins in *O. parapolymorpha*. Orthologs were identified using blastp (<https://blast.ncbi.nlm.nih.gov/>). *HPODL_03666 and HPODL_02528 have been experimentally verified as cytosolic and mitochondrial alcohol dehydrogenases in *O. parapolymorpha*, respectively (77, 78).

| ***S. cerevisiae* protein** | **Annotation** | ***O. parapolymorpha* ortholog** | **E-value (blastp)** |
| --- | --- | --- | --- |
| Adh1 | Cytosolic alcohol dehydrogenase | HPODL_03666* | 0.0 |
| Adh3 | Mitochondrial alcohol dehydrogenase | HPODL_02528* | 0.0 |
| Mdh1 | Mitochondrial malate dehydrogenase | HPODL_00710 | 2*10^-174^ |
| Mdh2 | Cytosolic malate dehydrogenase | HPODL_02341 | 2*10^-83^ |
| Aat1 | Mitochondrial aspartate aminotransferase | HPODL_01819 | 2*10^-91^ |
| Aat2 | Cytosolic aspartate aminotransferase | HPODL_02844 | 0.0 |
| Dic1 | Mitochondrial dicarboxylate carrier | HPODL_04016 | 9*10^-112^ |
| Oac1 | Mitochondrial oxaloacetate carrier | HPODL_00744 | 5*10^-157^ |
| Odc1 / Odc2 | Mitochondrial oxodicarboxylate carrier | HPODL_01147 | 8*10^-144^ / 3*10^-140^ |
| Agc1 | Mitochondrial amino acid transporter | HPODL_03446 | 5*10^-122^ |

Table S4: Primers used in this study.

| **Name** | **Sequence (5’-3’)** | **Purpose** |
| --- | --- | --- |
| 12097 | TTAGAAAAACTCATCGAGCATC | Construction of pUD740 |
| 12098 | ATGGGTAAGGAAAAGACTCAC | Construction of pUD740 |
| 12099 | ATCAGCATCCATGTTGGAATTTAATCGCGGCCTCGAAACGTGAGTCTTTTCCTTACCCATTTTGTAATTAAAACTTAGATTAGATTGCTATGC | Construction of pUD740 |
| 12100 | CCTTGCCAACAGGGAGTTC | Construction of pUD740, pUD801 |
| 12095 | ACCTTAAGTGCATATGCCGTATAAGGGAAACTCAAAGAACTGGCATCGCAAAAATGAAAACGTTCAGGGTAATATATTTTAAC | Construction of pUD740 |
| 12096 | ATTGATAATCCTGATATGAATAAATTGCAGTTTCATTTGATGCTCGATGAGTTTTTCTAAATAAAGCAATCTTGATGAGG | Construction of pUD740 |
| 10426 | AATCTATAATCAGTCCATAGTCAACAAGAGCC | Construction of pUD740, pUD803 |
| 10427 | TTTTCATTTTTGCGATGCCAGTTCTTTG | Construction of pUD740, pUD803 |
| 12093 | TAATACGACTCACTATAGGGCGAATTGGCGGAAGGCCGTCAAGGCCGCATCAACGAGCTCTGTGCCTAGGATTATGTCCTG | Construction of pUD740 |
| 12094 | GAGATCACTAAACGATATCAACTTTGAGGGCTCTTGTTGACTATGGACTGATTATAGATTATAGTCTGATACTCAGAAAGATCGAAG | Construction of pUD740 |
| 12101 | AGGCTGTCAATAATTTCGTTTTGAGCCTCCATGTCTCTGAAGAACTCCCTGTTGGCAAGGATCGTGCTCAAAGCCTACAAG | Construction of pUD740 |
| 12102 | CAGGTTTCCCGACTGGAAAGCGGGCAGTGAGCGGAAGGCCCATGAGGCCCAGAGGGTACCCAAATTATAGTACTTGTTTAGCTGCC | Construction of pUD740 |
| 12103 | GAGCTCGTTGATGCGGC | Construction of pUD740, pUD801, pUD802, pUD803, pUD1035, pUD1036 |
| 12104 | GGTACCCTCTGGGCCTC | Construction of pUD740, pUD801, pUD802, pUD803, pUD1035, pUD1036 |
| 4377 | CAGTATTAGTCGCCGCTTAG | Construction of pUD801 |
| 14385 | CAGGTTTCCCGACTGGAAAGCGGGCAGTGAGCGGAAGGCCCATGAGGCCCAGAGGGTACCTTGAGCTGGAGCCCAGCG | Construction of pUD801 |
| 14386 | AGGCTGTCAATAATTTCGTTTTGAGCCTCCATGTCTCTGAAGAACTCCCTGTTGGCAAGGAATCTAAGATAGTAAAATGTTCAGGTGGAAGTTTG | Construction of pUD801 |
| 14387 | ATATGCACTTAAGGTTTGATTTTGCTAATTAAAGGCGTGCCTAAGCGGCGACTAATACTGCGCTTTGGCCCAGATCAAGCC | Construction of pUD801 |
| 14388 | TAATACGACTCACTATAGGGCGAATTGGCGGAAGGCCGTCAAGGCCGCATCAACGAGCTCGGACGCACCCCTGGTTTATGC | Construction of pUD801 |
| 11065 | ACTATATGTGAAGGCATGGCTATGG | Construction of pUD802 |
| 11133 | GTTGAACATTCTTAGGCTGGTC | Construction of pUD802 |
| 14389 | CAGGTTTCCCGACTGGAAAGCGGGCAGTGAGCGGAAGGCCCATGAGGCCCAGAGGGTACCTATCCCTAGAATTGAGGCTAATTCGGCTTC | Construction of pUD802 |
| 14390 | GTGCCTATTGATGATCTGGCGGAATGTCTGCCGTGCCATAGCCATGCCTTCACATATAGTTATTTAGGATATTTCGAAAAAATACTGCCGACAAAC | Construction of pUD802 |
| 14391 | CACCTTTCGAGAGGACGATGCCCGTGTCTAAATGATTCGACCAGCCTAAGAATGTTCAACCACTTGAAGCGTCTTGAGAGATACAAGAAC | Construction of pUD802 |
| 14392 | TAATACGACTCACTATAGGGCGAATTGGCGGAAGGCCGTCAAGGCCGCATCAACGAGCTCCGCTGGTGATTTCTCTGGGAGAC | Construction of pUD802 |
| 14383 | AGCATCACCAGGGACTGATGTTCTGTATCTGTAGGCTGTATCATCTAAGGTAGTACCCATTGTTGATTATGTTTTTAAGAACTACTC | Construction of pUD803 |
| 14384 | TATGATGGAACAGCATCTGACGGTGAACAGGCCTTGTATATGTCTATGCCTTGCCCTTAAGCGGTTTTGTATAACTAAATAATATTGG | Construction of pUD803 |
| 14381 | ATGGGTACTACCTTAGATG | Construction of pUD803 |
| 14382 | TTAAGGGCAAGGCATAGAC | Construction of pUD803 |
| 14393 | CAGGTTTCCCGACTGGAAAGCGGGCAGTGAGCGGAAGGCCCATGAGGCCCAGAGGGTACCCCAAAGAGCCCAAATGTCTATATTTGAAAGG | Construction of pUD803 |
| 14394 | GAGATCACTAAACGATATCAACTTTGAGGGCTCTTGTTGACTATGGACTGATTATAGATTACTGTGCCACTGTGCGTACC | Construction of pUD803 |
| 14395 | ACCTTAAGTGCATATGCCGTATAAGGGAAACTCAAAGAACTGGCATCGCAAAAATGAAAAACAGCTTCAACACTCTTCCGACAG | Construction of pUD803 |
| 14396 | TAATACGACTCACTATAGGGCGAATTGGCGGAAGGCCGTCAAGGCCGCATCAACGAGCTCTATTGGCAACAAACTCCGATCGGATATTG | Construction of pUD803 |
| 14803 | CAGGTTTCCCGACTGGAAAGCGGGCAGTGAGCGGAAGGCCCATGAGGCCCAGAGGGTACCTACAAGAACTGTTTGCTGAGCTTGAG | Construction of pUD1035 |
| 14804 | CCCTGAGCTGCGCACGTCAAGACTGTCAAGGAGGGTATTCTGGGCCTCCATGTCGCTGGCGCATCCCTGGCGGAAAAATT | Construction of pUD1035 |
| 14805 | TTAAGTGCGCAGAAAGTAATATCATGCGTCAATCGTATGTGAATGCTGGTCGCTATACTGTCCAACTTATGCCAACTTCGATCATC | Construction of pUD1035 |
| 14806 | TAATACGACTCACTATAGGGCGAATTGGCGGAAGGCCGTCAAGGCCGCATCAACGAGCTCGATAATATTTTGGGAGAGGAGGATTTGATATGG | Construction of pUD1035 |
| 3242 | CAGTATAGCGACCAGCATTC | Construction of pUD1035, pUD1036 |
| 8439 | GCCAGCGACATGGAGGCCCAGAATAC | Construction of pUD1035, pUD1036 |
| 14807 | CAGGTTTCCCGACTGGAAAGCGGGCAGTGAGCGGAAGGCCCATGAGGCCCAGAGGGTACCTTGGTACGGCCAGCTAAACG | Construction of pUD1036 |
| 14808 | CCCTGAGCTGCGCACGTCAAGACTGTCAAGGAGGGTATTCTGGGCCTCCATGTCGCTGGCTGCATTTCGGGAAACGTGAAC | Construction of pUD1036 |
| 14809 | TTAAGTGCGCAGAAAGTAATATCATGCGTCAATCGTATGTGAATGCTGGTCGCTATACTGTGCCGCCCGCCTG | Construction of pUD1036 |
| 14810 | TAATACGACTCACTATAGGGCGAATTGGCGGAAGGCCGTCAAGGCCGCATCAACGAGCTCTTTTTCCAATCTATATCTTTATTTTTCATCAT | Construction of pUD1036 |
| 2908 | GGATTGGGTGTGATGTAAGGATTCGC | Diagnostic PCR of pUD801 |
| 12612 | CAACAACATCACTCCATCTC | Diagnostic PCR of pUD801 |
| 1642 | TTTCCCAGTCACGACGTTG | Diagnostic PCR of pUD801, pUD802, pUD803, pUD1035, pUD1036 |
| 3983 | AGACCGATACCAGGATCTTG | Diagnostic PCR of pUD801 |
| 2457 | CGCACGTCAAGACTGTCAAG | Diagnostic PCR of pUD802 |
| 12616 | CGAGTCAGTGAGCGAGGAAG | Diagnostic PCR of pUD801, pUD802, pUD803, pUD1035, pUD1036 |
| 1781 | TACTCGCCGATAGTGGAAAC | Diagnostic PCR of pUD802 |
| 10459 | TGGCGGTTACTCTAAAGACG | Diagnostic PCR of pUD803 |
| 10458 | GAGGAGCCGGTCATTTATGG | Diagnostic PCR of pUD803 |
| 1409 | TATTCTGGGCCTCCATGTCGCTGG | Diagnostic PCR of pUD1035, pUD1036 |
| 4662 | GACATCATCTGCCCAGATGC | Diagnostic PCR of pUD1035, pUD1036 |
| 14929 | CATGATCCATGGGCAGCCATCACCATCACCATCACGGCAGCTCTGCCCAGCGCAAGTC | Construction of pTrc99A-NDH2-1 |
| 14931 | CATCAGCCCGGGCTACTCGTTAGTCAAGTCCCTACC | Construction of pTrc99A-NDH2-1 |
| 16075 | CATGATCCATGGGCAGCCATCACCATCACCATCACGGCAGCCAACGACAACTAGCGTCAGTGG | Construction of pTrc99A-NDH2-2 |
| 16076 | CATCAGCCCGGGTTAGTCTTTGGAGCAGTCGCGAC | Construction of pTrc99A-NDH2-2 |
| 16077 | CATGATCCATGGGCAGCCATCACCATCACCATCACGGCAGCTTCCCATCAATAATAAAGGTTGGCC | Construction of pTrc99A-NDH2-3 |
| 16078 | CATCAGCCCGGGTTAAACAGTCAAGATATCCCTACC | Construction of pTrc99A-NDH2-3 |
| 10742 | CCGTGCGCCTCAACACTATC | Diagnostic PCR of genomic *NDH2-1* disruption |
| 10743 | GACCTCCTTGGCGATGGATG | Diagnostic PCR of genomic *NDH2-1* disruption |
| 10744 | CGCCTGTTTGGACACTCCTC | Diagnostic PCR of genomic *NDH2-2* disruption |
| 10745 | TTCTGGCAACAGTCTGGATG | Diagnostic PCR of genomic *NDH2-2* disruption |
| 10746 | TGGTTGCACGGTAACTATGG | Diagnostic PCR of genomic *NDH2-3* disruption |
| 10774 | TTATGCCGTCTCAGGTCTCACAGCCAGTGTTCCTTAATCAAGGATACC | Diagnostic PCR of genomic *NDH2-3* disruption |
| 6816 | ATTCCGACTCGTCCAACATC | Amplification of left split-marker fragment from pUD801 for deletion of *NDH2-1* |
| 14397 | TTGAGCTGGAGCCCAG | Amplification of left split-marker fragment from pUD801 for deletion of *NDH2-1* |
| 12565 | AAAGGTAGCGTTGCCAATG | Amplification of right split-marker fragment from pUD801 for deletion of *NDH2-1* |
| 14398 | GGACGCACCCCTGG | Amplification of right split-marker fragment from pUD801 for deletion of *NDH2-1* |
| 14399 | TATCCCTAGAATTGAGGCTAATTCG | Amplification of left split-marker fragment from pUD802 for deletion of *NDH2-2* |
| 14400 | GCTCGAAGTAGCGCGTC | Amplification of left split-marker fragment from pUD802 for deletion of *NDH2-2* |
| 14401 | ATCTCCCGCCGTGC | Amplification of right split-marker fragment from pUD802 for deletion of *NDH2-2* |
| 14402 | CGCTGGTGATTTCTCTGGG | Amplification of right split-marker fragment from pUD802 for deletion of *NDH2-2* |
| 14403 | ATCATATAAAGCAGTATCTAAACCAC | Amplification of left split-marker fragment from pUD803 for deletion of *NDH2-3* |
| 14404 | CCAAAGAGCCCAAATGTCTA | Amplification of left split-marker fragment from pUD803 for deletion of *NDH2-3* |
| 14405 | TATTGGCAACAAACTCCG | Amplification of right split-marker fragment from pUD803 for deletion of *NDH2-3* |
| 14406 | CCACCGGTGATGGATTTAC | Amplification of right split-marker fragment from pUD803 for deletion of *NDH2-3* |
| 14811 | TACAAGAACTGTTTGCTGAGCTTGAG | Amplification of left split-marker fragment from pUD1035 for deletion of *GUT2* |
| 14812 | GATAATATTTTGGGAGAGGAGGATTTG | Amplification of right split-marker fragment from pUD1035 for deletion of *GUT2* |
| 15884 | GTACGGCCAGCTAAACGC | Amplification of left split-marker fragment from pUD1036 for deletion of *NUBM* |
| 15887 | GTTCCGGTAACAAGAGGAC | Amplification of right split-marker fragment from pUD1036 for deletion of *NUBM* |
| 15885 | GTGCTTGTAGCCGGCTG | Amplification of left split-marker fragment from pUD1036 for deletion of *NUBM*; amplification of left split-marker fragment from pUD1035 for deletion of *GUT2*; diagnostic PCR of *ΔNUBM:: pat* deletion; diagnostic PCR of *ΔGUT2:: pat deletion* |
| 15886 | TCTGCGACATCGTCAATC | Amplification of right split-marker fragment from pUD1036 for deletion of *NUBM*; amplification of right split-marker fragment from pUD1035 for deletion of *GUT2*; diagnostic PCR of *ΔNUBM:: pat* deletion; diagnostic PCR of *ΔGUT2:: pat deletion* |
| 14465 | TACGTCGACTAACAAGGAGC | Diagnostic PCR of *ΔNDH2-1:: kanR* deletion |
| 14466 | GCGGTCTCGACAGCTTTC | Diagnostic PCR of *ΔNDH2-1:: kanR* deletion |
| 4047 | AACTCACCGAGGCAGTTCCATAG | Diagnostic PCR of *ΔNDH2-1:: kanR* deletion |
| 2653 | GGCAATCAGGTGCGACAATC | Diagnostic PCR of *ΔNDH2-1:: kanR* deletion |
| 14467 | CTAGTGAAATTCCACTCTATAAATCGATCAT | Diagnostic PCR of *ΔNDH2-2:: hph* deletion |
| 14468 | AAAGTCGGAGAGTGTTTTAGACC | Diagnostic PCR of *ΔNDH2-2:: hph* deletion |
| 7864 | TCTGGGCAGATGATGTCGAG | Diagnostic PCR of *ΔNDH2-2:: hph* deletion |
| 8411 | CGTTGAATTGTCCCCACG | Diagnostic PCR of *ΔNDH2-2:: hph* deletion |
| 14469 | ATGTACAGGAATCTATATTTTTAAACAGTCAAG | Diagnostic PCR of *ΔNDH2-3:: NatR* deletion |
| 14470 | AACTATGGATCTTTGAAAAAAAAAGCAAAC | Diagnostic PCR of *ΔNDH2-3:: NatR* deletion |
| 11197 | ATGGGTTCCTGACTGACTAC | Diagnostic PCR of *ΔNDH2-3:: NatR* deletion |
| 11202 | CACACCGGAAATCAAGGCAT | Diagnostic PCR of *ΔNDH2-3:: NatR* deletion |
| 15628 | GACAAGAACGACGCGCTTTCGG | Diagnostic PCR of *ΔGUT2:: pat* deletion |
| 15629 | ACGGCCAATATACTGCACGACC | Diagnostic PCR of *ΔGUT2:: pat* deletion |
| 15630 | ACTTGAATATGCGGCCTGCAGC | Diagnostic PCR of *ΔNUBM:: pat* deletion |
| 15631 | CTGTCCGCCGTATCATCCTTCC | Diagnostic PCR of *ΔNUBM:: pat* deletion |
